# Molecular Origin of Correlated Bath Effects in Photoinduced Charge Transfer Dynamics in Polar Solvents

**DOI:** 10.1021/acs.jpclett.5c04090

**Published:** 2026-03-16

**Authors:** Xiang Sun, Zengkui Liu

**Affiliations:** † Division of Arts and Sciences, 447103 NYU Shanghai, 567 West Yangsi Road, Shanghai 200124, China; ‡ Department of Chemistry, New York University, New York, New York 10003, United States; ¶ NYU-ECNU Center for Computational Chemistry at NYU Shanghai, 3663 Zhongshan Road North, Shanghai 200062, China; § Center for Data Science, NYU Shanghai, 567 West Yangsi Road, Shanghai 200124, China; ∥ State Key Laboratory of Precision Spectroscopy, East China Normal University, Shanghai 200062, China

## Abstract

Nonadiabatic dynamics in the condensed phase often involve correlated environments shared by multiple electronic states, challenging the traditional isolated bath assumption. We investigate these effects using the multistate harmonic (MSH) model and atomistic Hamiltonian applied to photoinduced charge transfer in a trimer consisting of a methylperylene donor and two tetracyanoethylene acceptors dissolved in a polar solvent. We propose a geometric metric based on the angular relationship of reorganization energies between transitions sharing an initial state to quantify bath correlation. Our analysis identifies distinct regimes: a correlated bath where synchronized energy gap fluctuations facilitate competing reactions, and an anticorrelated bath where fluctuations favoring one reaction suppress the other. These energetic correlations are modulated by molecular conformation and charge distribution, specifically through changes in dipole moments and solvent-accessible surface area. This study provides a connection between the energetic perspective of environmental correlations and the molecular details governing nonadiabatic dynamics in polar solvents.

## Graphical abstract

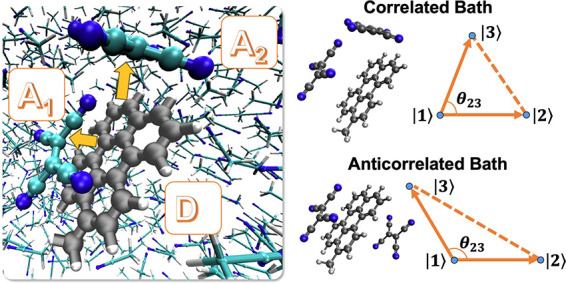

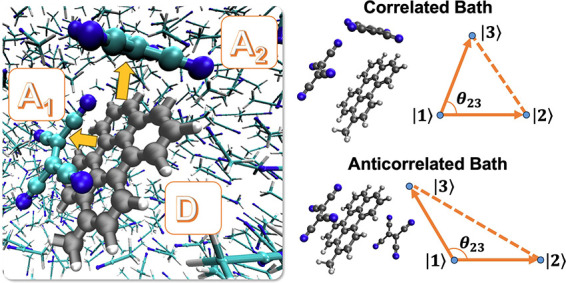

Nonadiabatic dynamics plays a fundamental role in the photophysical and photochemical processes governing solar energy conversion, ranging from artificial photovoltaic cells to natural photosynthetic systems.^1–9^ The complex, intertwined nature of electronic and nuclear degrees of freedom (DOF) in these systems requires approaches that go beyond the widely adopted Born–Oppenheimer approximation underlying modern quantum chemistry. Consequently, there is a rich history of developing nonadiabatic dynamics methods for complex molecular systems in the condensed phase, aiming to elucidate the mutual influence of electronic and nuclear DOF within effective model or atomistic molecular Hamiltonians.^10–12^ In standard system-bath models, the relevant electronic states typically constitute the basis for the system Hamiltonian, while the nuclear DOF consisting of intramolecular and intermolecular vibrations form that for the bath Hamiltonian, with system-bath coupling mediating the nonadiabatic dynamics.^13–19^ A widely used example is the Frenkel exciton model for excitation energy transfer (EET),^20,21^ which traditionally relies on the “isolated bath assumption”: that each chromophore couples to its own local environment, and that these local baths are statistically independent.^22–28^

However, the isolated bath assumption breaks down when chromophores are in close proximity, sharing a common solvent environment, or when charge transfer (CT) states form between moieties embedded in the same solvation environment.^23–25,27,29–31^ In such scenarios, one expects significant shared or correlated bath effects to influence the nonadiabatic molecular dynamics (NAMD). Theoretical treatments of solvation dynamics have long recognized that solvent motions are not merely independent local fluctuations but organize into collective modes. Seminal work by Stratt and collaborators utilizing instantaneous normal mode (INM) theory demonstrated that the solvent shell possesses specific symmetries that can selectively couple to solute electronic transitions.^32–35^ These studies highlighted that the solvent response is inherently collective, with solvent motions capable of promoting or suppressing specific reaction pathways depending on how the solvent’s modal symmetry aligns with the electronic transition dipoles.

The dynamical consequences of such environmental correlations have been a subject of significant theoretical inquiry. Model studies by Strümpfer and Schulten^24^ demonstrated that correlated bath fluctuations can modify energy transfer efficiency in photosynthetic complexes. Similarly, Dutta et al.^36^ showed that spatial and temporal correlations critically shape excitonic spectral line shapes. However, characterizing the magnitude of these effects in realistic systems remains nontrivial. For instance, in atomistic molecular dynamics simulations of the light-harvesting II complex, Olbrich and Kleinekathöfer^37^ found that the cross-correlations between the excitation energy fluctuations of different pigments were negligible. This contrast suggests that the manifestation of bath correlation is highly sensitive to the molecular geometry, the nature of the electronic states, and the specific correlation functions employed to quantify the environment. For instance, a recent study on the EET of two nonfullerene acceptor Y6 molecules in chloroform demonstrated that the bath transitions from correlated to uncorrelated as the intermolecular distance increases.^38^ Similarly, photoinduced CT dynamics in a carotenoid-porphyrin-fullerene (donor-bridge-acceptor) triad dissolved in tetrahydrofuran reveal that the locally excited ππ* state and the various CT states share a common solvent environment, leading to conformation-dependent correlated or even anticorrelated bath effects.^31,38,39^

It appears that correlated bath effects in nonadiabatic processes are dictated not merely by physical proximity or shared environments, but fundamentally by how this shared environment couples to the multiple electronic states. Broadly, bath correlation describes a regime where the environments of distinct electronic states cannot be treated independently. Yet, the precise definition of a “correlated bath” varies in the literature and often lacks a clear, quantitative molecular picture. In this Letter, we focus on establishing a rigorous description of correlated bath effects in nonadiabatic dynamics and clarifying the underlying molecular physical picture.

The recently developed multistate harmonic (MSH) model^31,38,39^ provides an ideal framework for this task, allowing for an unambiguous definition of the degree of bath correlation. In contrast to standard excitonic models that partition the environment into independent local baths, the MSH framework employs a “globally shared bath”, where a single set of global normal modes couples to all electronic states simultaneously. This globally shared bath is parametrized from realistic anharmonic atomistic simulations to systematically satisfy all pairwise reorganization energy constraints. This globally shared bath is rigorously defined within an extended (*F* – 1)-dimensional nuclear space for an *F*-state system. The MSH model’s global bath naturally encompasses the full spectrum of environmental correlations, ranging from correlated to anticorrelated bath regimes, and recovers the uncorrelated or isolated bath limit as a specific critical case.^38^

To quantify the bath correlation, one must consider at least three electronic states and two distinct electronic transitions, for example, |1⟩→|2⟩ and |1⟩→|3⟩. In the extended nuclear space, the square roots of the pairwise reorganization energies for these transitions form a triangle, allowing the correlation to be defined by the angle θ_23_ between the transitions:

$$\cos\theta_{23}=\frac{E_{r}^{\left(12\right)}+E_{r}^{\left(13\right)}-E_{r}^{\left(23\right)}}{2\sqrt{E_{r}^{\left(12\right)}E_{r}^{\left(13\right)}}}$$ 1

Here, *E*_*r*_^(12)^, *E*_*r*_^(13)^, and *E*_*r*_^(23)^ represent the reorganization energies for the transitions |1⟩↔|2⟩, |1⟩↔|3⟩, and |2⟩↔|3⟩, respectively. These values are determined directly from the energy gap fluctuations in all-atom simulations via:

$$E_{r}^{\left(XY\right)}=\frac{\sigma_{XY}^{2}}{2k_{B}T}$$ 2

In this expression, σ_*XY*_^2^ = ⟨*U*_*XY*_^2^⟩ – ⟨*U*_*XY*_⟩^2^ is the variance of the energy gap *U*_*XY*_ = *V*_*X*_ – *V*_*Y*_, and *k*_*B*_ *T* is the thermal energy with Boltzmann constant *k*_*B*_ and temperature *T*. For EET, |1⟩ is typically selected as the ground state while |2⟩ and |3⟩ are locally excited states; for photoinduced CT, |1⟩ may be a locally excited state while |2⟩ and |3⟩ are two CT states between different molecules or of distinguished CT character. Importantly, the MSH model preserves the exact reorganization energy values obtained from the all-atom Hamiltonian.^39^

To provide a physical interpretation of this geometric metric, it is instructive to consider the distinct regimes of bath correlation. Mathematically, cosθ_23_ corresponds to the normalized cross-correlation coefficient between the energy gap fluctuations of the two transitions. When θ_23_ = 90°, the cosine is zero, implying that the fluctuations of the energy gaps *U*_12_ and *U*_13_ are statistically uncorrelated. In the MSH framework, this signifies that the equilibrium nuclear displacements for the two transitions are orthogonal in the extended-dimensional nuclear space; physically, this corresponds to a scenario where the bath motions coupled to one transition are entirely independent of those coupled to the other, as often observed in EET between spatially well-separated chromophores.^38^ Deviations from orthogonality indicate bath correlation effects: an acute angle (θ_23_ < 90°) implies a positive correlation, where solvent fluctuations that stabilize state |2⟩ (relative to |1⟩) simultaneously stabilize state |3⟩. Conversely, an obtuse angle (θ_23_ > 90°) implies a statistical anticorrelation, where solvent motions favoring one transition energetically penalize the other. We emphasize that this classification is transition-specific; a globally shared bath may be correlated for one pair of transitions while being anticorrelated for another within the same MSH model.

To this end, we propose using the angle θ_23_ = θ_CT1,CT2_ as a quantitative metric to categorize the degree of bath correlation for the transitions of interest (e.g., EX → CT1 and EX → CT2). Specifically: (1) θ_23_ < 90° indicates a correlated bath; (2) θ_23_ > 90° indicates an anticorrelated bath; and (3) θ_23_ = 90° indicates an uncorrelated (independent) bath.^38^ This geometric definition highlights that the degree of bath correlation is an intrinsic property of the system’s energy gap fluctuations, dependent on the interplay of all three pairwise reorganization energies.

In this work, we investigate correlated bath effects in nonadiabatic dynamics by focusing on a donor–acceptor-acceptor (DA_1_A_2_) molecular trimer. This system comprises one methylperylene (MPe) donor and two tetracyanoethylene (TCNE) acceptor molecules dissolved in acetonitrile (ACN) solvent at 300 K.^38,40–43^ Our approach goes beyond qualitative descriptions of collective solvent modes by providing: (a) a rigorously defined, model-independent geometric metric (θ_23_) for classifying bath correlation regimes; (b) the integration of this metric into the MSH formalism, which preserves atomistic bath correlations within a reduced-dimensionality framework; (c) a concrete molecular interpretation of the metric that extends beyond abstract collective solvent modes; and (d) a systematic model construction strategy that disentangles bath correlation effects from those driven by the system Hamiltonian. The intermolecular photoinduced CT process initiates with the formation of an excitonic (EX) state localized on the MPe molecule (D*A_1_A_2_). This is followed by electronic transitions to two distinct CT states, denoted as CT1 and CT2, corresponding to charge transfer from the MPe donor to the first (D^+^A_1_^–^A_2_) and second (D^+^A_1_A_2_^–^) TCNE acceptor molecules, respectively.

Figure 1(a1–c1) depicts three selected conformations of the trimer: the gas-phase optimized structure (R0); a modified structure (R1) where the first TCNE molecule (A_1_) is displaced 3 Å away from the donor relative to R0; and a sandwich-like structure (R2) where the two TCNE acceptors are positioned on opposite sides of the MPe donor. Our analysis centers on the transitions of interest, EX → CT1 and EX → CT2, with the corresponding reorganization energy relationships illustrated in Figure 1(a2–c2). Table 1 lists the reorganization energies for all state pairs across the three conformations. Based on the angular parameter θ_23_ listed in Table 1, which is computed using these reorganization energies, it is evident that conformations R0 and R1 exhibit a correlated bath, whereas conformation R2 displays an anticorrelated bath.

**Figure 1:**
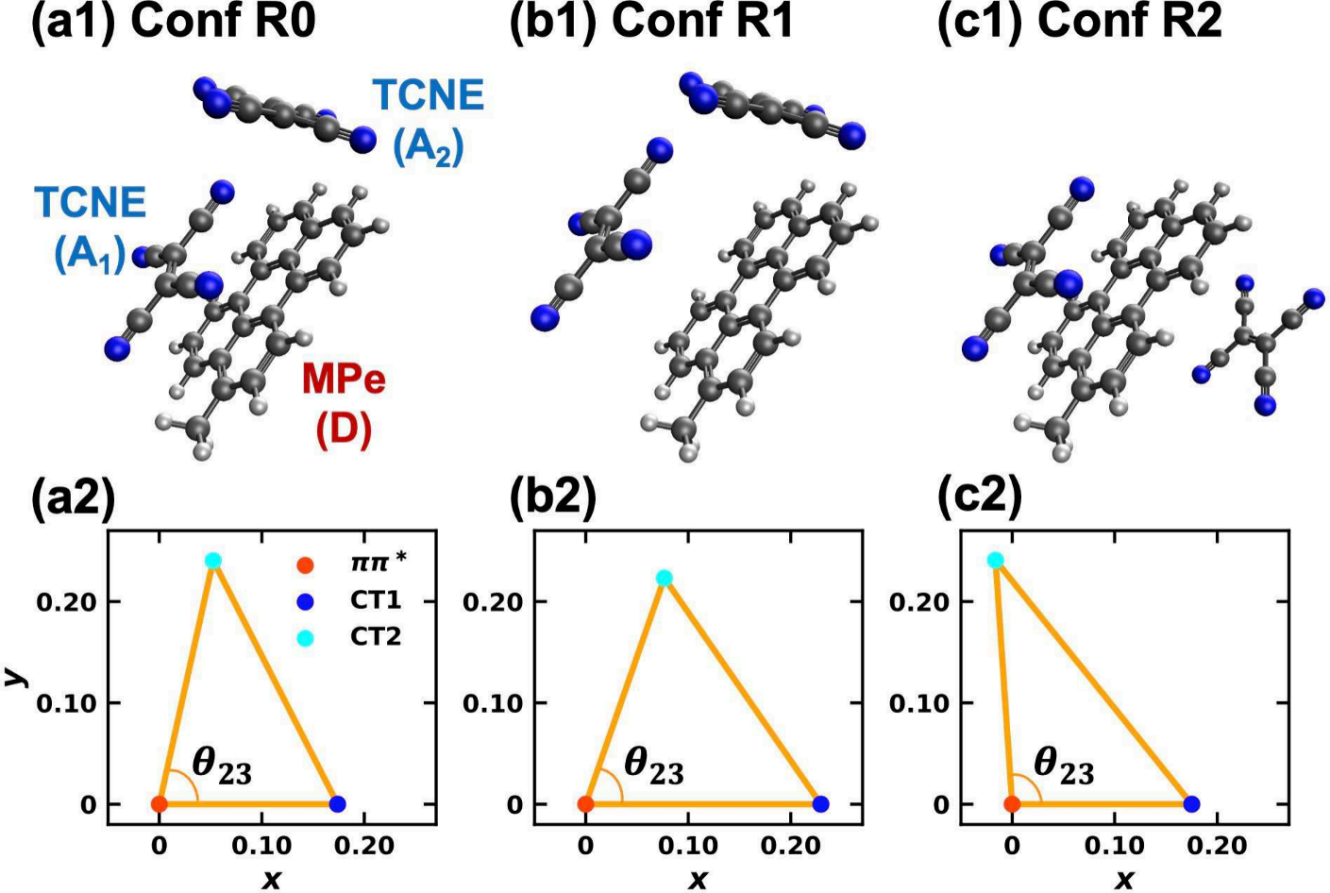
Conformations (top) and the triangles (bottom) formed by the three excited-state potential energy surface minima of the MPe/2TCNE (DA_1_A_2_) trimer in Scheme 1: (a) the gas-phase optimized conformation R0 dissolved in polar solvent acetonitrile forming a correlated bath for the photoinduced charge transfer process; (b) conformation R1 obtained by moving TCNE (A_1_) 3 Å further from MPe (D) based on conformation R0, forming a correlated bath; (c) conformation R2 obtained by rotating TCNE (A_2_) from conformation R0 to the opposite side of MPe (D), forming an anticorrelated bath. The *x*, *y* axes in the bottom panels scale with the square root of the reorganization energies in atomic units.

**Table 1:** All-Atom Parameters of the Three Conformations (R0, R1, R2) of the MPe/2TCNE Trimer Obtained Using Scheme 1 [S1] and Scheme 2 [S2] with *j* = 1, 2, 3 Corresponding to EX, CT1, CT2 States, Respectively^a^

	R0	R1 [S1]	R1 [S2]	R2 [S1]	R2 [S2]
Γ_12_	–74.3	22.8	–74.3	–54.5	–74.3
Γ_13_	20.4	43.8	20.4	–8.5	20.4
Γ_23_	10.3	240	10.3	1.5	10.3
*E*_*r*_^(12)^	0.823	1.432	1.295	0.835	0.812
*E*_*r*_^(13)^	1.648	1.515	1.740	1.586	1.660
*E*_*r*_^(23)^	1.934	1.990	2.222	2.444	2.750
θ_12_	39.12	53.35	47.30	34.62	32.66
θ_13_	63.22	55.62	58.41	51.52	50.47
θ_23_	77.66	71.03	74.29	93.86	96.87
*E*_1_	3.078	3.069	3.078	3.084	3.087
*E*_2_	2.164	2.022	2.164	2.532	2.164
*E*_3_	2.377	2.202	2.377	2.153	2.377
*W*_1_	3.343	2.647	3.350	3.420	3.351
*W*_2_	1.534	0.377	0.956	1.267	1.475
*W*_3_	–0.464	–0.138	–0.272	–0.030	–0.405
|Δ**μ**_12_|	16.8	27.6	25.6	17.0	16.6
|Δ**μ**_13_|	32.6	29.8	32.6	30.2	31.4
|Δ**μ**_23_|	32.8	30.4	33.6	47.1	48.0
α_12,13_	75.6	65.0	69.4	173.4	178.0
α_23,13_	29.8	52.8	45.5	2.4	0.7
α_12,23_	105.6	117.7	114.9	175.7	178.4

a Here, *E*_*j*_ is the excitation energy (in eV) of state *j*, Γ_*jk*_ is the electronic coupling (in eV) between states *j* and *k*, *E*_*r*_^(*jk*)^ is the reorganization energy (in eV) between states *j* and *k*, θ_*jk*_ is the angle (in degree) of the PES triangle defined in eq 1, *W*_*j*_ is the energy correction (in eV) to the FF of state *j*, Δ**μ**_*jk*_ = **μ**_*j*_ – **μ**_*k*_ is the permanent dipole difference between states *j* and *k* (in Debye), and α_*jk*,*mn*_ is the angle (in degree) between Δ**μ**_*jk*_ and Δ**μ**_*mn*_.

To describe the nonadiabatic dynamics, we employ the general *F*-state Hamiltonian

$$H=\left(\begin{array}{llll} H_{1} & \Gamma_{12} & \cdots & \Gamma_{1F}\\ \Gamma_{21} & H_{2} & \cdots & \Gamma_{2F}\\ \vdots & \vdots & \ddots & \vdots \\ \Gamma_{F1} & \Gamma_{F2} & \cdots & H_{F} \end{array}\right)$$ 3

where the diagonal terms represent the nuclear Hamiltonian of the *j*th electronic state (*j* = 1, ···, *F*), given by *H*_*j*_ = **P** ^2^/2 + *V*_*j*_(**R**). Here, **R** and **P** are the mass-weighted nuclear positions and momenta, and *V*_*j*_(**R**) is the potential energy surface (PES) of the *j*th electronic state. The off-diagonal terms Γ_*jk*_ (*j*≠*k*) describe the electronic coupling between states *j* and *k*. For the all-atom multistate Hamiltonian, we construct the PES of the *F* states using a modified general Amber force field (GAFF), where the bonded interaction parameters are kept the same according to GAFF. However, the atomic charges and excitation energies of different electronic states are parametrized via the electronic structure calculations, as detailed in the Supporting Information. This potential of the *j*th state is expressed as^39^

$$V_{j}\left(R\right)=V_{j}^{\mathrm{FF}}\left(R\right)+W_{j}\left(r^{\mathrm{tri}}\right)$$ 4

Here, *V*_*j*_^FF^(**R**) represents the force field (FF) potential energy of the *j*th state. The term *W*_*j*_(**r** ^tri^) = *E*_*j*_(**r** ^tri^) – *V*_*j*_^FF^(**r** ^tri^) acts as an energy correction that incorporates the excitation energy *E*_*j*_(**r** ^tri^) obtained from electronic structure calculations for the trimer geometry **r** ^tri^, and the all nuclear positions **R** includes the solute trimer configuration **r** ^tri^ and the solvent configuration. This correction explicitly removes the double-counted intramolecular trimer energy in the FF term *V*_*j*_^FF^(**r** ^tri^), which has been already included in the excitation energy *E*_*j*_(**r** ^tri^) evaluated at a higher quantum mechanical level. For more details, refer to refs 31 and 44.

Alternatively, within the MSH model Hamiltonian, the PES of the *j*th state takes the form^39^

$$V_{j}\left(R\right)=\overset{F-1}{\underset{a=1}{\sum }}\overset{N}{\underset{i=1}{\sum }}\left[\frac{1}{2}\omega_{i}^{2}{\left(R_{a,i}-S_{i}^{\left(aj\right)}\right)}^{2}\right]+\varepsilon_{j}$$ 5

where the nuclear DOF span an extended (*F* – 1)-dimensional space. The coordinate *R*_*a*,*i*_ corresponds to the *i*th normal mode with frequency ω_*i*_ (*i* = 1, ···, *N*) within the *a*th nuclear subspace (*a* = 1, ···, *F* – 1). The parameter *S*_*i*_^(*aj*)^ defines the equilibrium shift, subject to the constraint that *S*_*i*_^(*aj*)^ ≡ 0 when *a* ≥ *j*. The set of discretized frequencies {ω_*i*_} is determined by the spectral density, which is derived directly from the energy-gap time correlation function calculated in the all-atom simulations.^39^ A fundamental requirement of the MSH model is that it must simultaneously satisfy all pairwise reorganization energies derived from all-atom simulations. This constraint is expressed as

$$E_{r}^{\left(XY\right)}=\overset{F-1}{\underset{a=1}{\sum }}\overset{N}{\underset{i=1}{\sum }}\frac{1}{2}\omega_{i}^{2}{\left(S_{i}^{\left(aX\right)}-S_{i}^{\left(aY\right)}\right)}^{2}$$ 6

The energy minima parameters, {*ε*_*j*_}, and their differences, defined as the reaction free energies Δ*E* ^(*XY*)^, are determined from the averaged energy gap and the reorganization energy via

$$\varepsilon_{Y}-\varepsilon_{X}=\Delta E^{\left(XY\right)}=-E_{r}^{\left(XY\right)}-\left\langle U_{XY}\right\rangle$$ 7

The MSH model Hamiltonian can be cast into system-bath form, *H* = *H*_S_ + *H*_B_ + *H*_BS_. The system Hamiltonian is given by

$$\begin{array}{rl} H_{S}=\left(\begin{array}{llll} \varepsilon_{1} & \Gamma_{12} & \cdots & \Gamma_{1F}\\ \Gamma_{21} & \varepsilon_{2} & \cdots & \Gamma_{2F}\\ \vdots & \vdots & \ddots & \vdots \\ \Gamma_{F1} & \Gamma_{F2} & \cdots & \varepsilon_{F} \end{array}\right) & \end{array}$$ 8

and the sum of bath and system-bath coupling terms is expressed as

$$H_{\mathrm{BBS}}=H_{B}+H_{\mathrm{BS}}=\mathrm{diag}\left\{{Ṽ}_{1}\left(R\right),\cdot \cdot \cdot ,{Ṽ}_{F}\left(R\right)\right\}$$ 9

where

$${Ṽ}_{j}\left(R\right)=\overset{F-1}{\underset{a=1}{\sum }}\overset{N}{\underset{i=1}{\sum }}\frac{1}{2}\omega_{i}^{2}{\left(R_{a,i}-S_{i}^{\left(aj\right)}\right)}^{2}$$

represents the shifted harmonic potential surfaces in the extended nuclear space without the vertical shifts *ε*_*j*_.

To investigate the contributions of different Hamiltonian components to the nonadiabatic dynamics, we designed three distinct parametrization schemes for both the all-atom description and the MSH model construction.

- **Scheme 1 [S1]**: This scheme represents a fully consistent, conformation-specific parametrization.^39^ For each conformation (R0, R1, and R2), the all-atom Hamiltonian is constructed using electronic couplings ({Γ_*jk*_}), excitation energies ({*E*_*j*_}), and multistate force field (FF) parameters—including atomic charges and energy corrections ({*W*_*j*_}) that are calculated individually for that specific geometry. Subsequently, the MSH model parameters for each conformation, including electronic couplings, energy minima ({*ε*_*j*_}), mode frequencies ({ω_*i*_}), and equilibrium shifts ({*S*_*i*_^(*aj*)^}), are determined directly from the corresponding all-atom simulations.
- **Scheme 2 [S2]**: This scheme is designed to isolate the effect of solute geometry on the bath while keeping electronic parameters fixed to the reference state. For constructing the all-atom Hamiltonians of conformations R1 and R2, we utilize the electronic couplings, excitation energies, FF parameters, and atomic charges derived from the R0 conformation. However, the trimer geometry itself is changed to R1 or R2, which requires recalculating the energy corrections to the force fields. Correspondingly, the MSH models for R1 and R2 under Scheme 2 retain the electronic couplings and excitation energies from the R0 electronic structure calculations, while reparameterizing the energy minima and the combined bath/system-bath coupling terms (*H*_BBS_) based on all-atom simulations of the R1 and R2 conformations within this mixed parametrization.
- **Scheme 3 [S3]**: This scheme effectively combines the electronic system of the reference state with the full environmental bath of the target state. To construct the MSH models for conformations R1 and R2, we employ the system Hamiltonian *H*_S_ (including electronic couplings and energy minima) taken directly from the R0 MSH model. In contrast, the bath and system-bath coupling terms (*H*_BBS_) are adopted from the fully consistent MSH models [S1] of the respective R1 and R2 conformations.

Consequently, conformation R0 is described solely by Scheme 1, whereas conformations R1 and R2 utilize Schemes 1–3. Scheme 1 is the standard consistent approach for parametrizing both all-atom and MSH Hamiltonians, where electronic structure and molecular dynamics calculations are derived directly from the specific geometry of each conformation, particularly regarding the charge distributions of different electronic states. Conversely, Scheme 2 for conformations R1 and R2 employs the electronic couplings, excitation energies, and force field parameters from conformation R0. This design allows us to isolate the influence of the trimer’s geometric changes on the nonadiabatic dynamics. Scheme 3 acts as an intermediate bridge between Schemes 1 and 2. Comparing the R0 reference with Scheme 3 for a given conformation (R1 or R2) highlights the differences arising from the bath and system-bath coupling parameters, as their system Hamiltonians are identical. Furthermore, comparing Schemes 1 and 3 for the same conformation (R1 or R2) isolates the impact of the system Hamiltonian, specifically the electronic couplings and excitation energies, as the bath parameters remain constant. We included Table S2 in the Supporting Information to summarize the main features of these schemes.

Table 1 details the all-atom parameters for the three trimer conformations under Schemes 1 and 2, including the reorganization energies, and the magnitudes of dipole moment changes accompanying electronic transitions. Table 2 summarizes the MSH model parameters for these conformations across Schemes 1 through 3, alongside the Marcus rate constants for all potential excited-state electronic transitions. Table 3 lists the solvent-accessible surface area (SASA) of the trimer segments calculated via the Shrake-Rupley approach,^45^ and the minimal intermolecular distances between the donor and the acceptors. For a generic transition *X* → *Y*, the Marcus rate constant is defined as^46–48^

$$k_{X\to Y}=\frac{|\Gamma_{XY}{|}^{2}}{\hbar }\sqrt{\frac{\pi }{k_{B}TE_{r}^{\left(XY\right)}}}\;\exp\left[-\frac{{\left(\Delta E_{XY}+E_{r}^{\left(XY\right)}\right)}^{2}}{4k_{B}TE_{r}^{\left(XY\right)}}\right]$$ 10

and the corresponding activation energy is given by

$$E_{\mathrm{act}}^{\left(XY\right)}=\frac{{\left(\Delta E_{XY}+E_{r}^{\left(XY\right)}\right)}^{2}}{4E_{r}^{\left(XY\right)}}$$ 11

**Table 2:** MSH Parameters of the Three Conformations (R0, R1, R2) of the MPe/2TCNE Trimer Obtained Using Schemes 1–3 (S1–S3) with *j* = 1, 2, 3 Corresponding to EX, CT1, CT2 States, Respectively^a^

Model	R0	R1 [S1]	R1 [S2]	R1 [S3]	R2 [S1]	R2 [S2]	R2 [S3]
*ε*_1_	3.078	3.078	3.070	3.078	3.075	3.071	3.078
*ε*_2_	1.507	0.892	0.724	1.507	1.439	1.401	1.507
*ε*_3_	0.902	0.987	0.771	0.902	0.973	0.946	0.902
Γ_12_	–74.3	22.8	–74.3	–74.3	–54.5	–74.3	–74.3
Γ_13_	20.4	43.8	20.4	20.4	–8.5	20.4	20.4
Γ_23_	10.3	240	10.3	10.3	1.5	10.3	10.3
*E*_*r*_^(12)^	0.823	1.432	1.295	1.432	0.835	0.812	0.835
*E*_*r*_^(13)^	1.648	1.515	1.740	1.515	1.586	1.660	1.586
*E*_*r*_^(23)^	1.934	1.990	2.222	1.990	2.444	2.750	2.444
θ_23_	77.66	71.03	74.29	71.03	93.86	96.87	93.86
*k*_1→2_	1.4 × 10^11^	1.8 × 10^11^	6.2 × 10^11^	6.8 × 10^13^	3.2 × 10^10^	1.6 × 10^10^	1.9 × 10^11^
*k*_2→1_	5.8 × 10^–16^	3.0 × 10^–26^	2.8 × 10^–24^	2.7 × 10^–13^	1.0 × 10^–17^	1.4 × 10^–18^	7.8 × 10^–16^
*k*_1→3_	1.1 × 10^12^	3.1 × 10^12^	1.5 × 10^12^	3.5 × 10^11^	1.9 × 10^11^	1.5 × 10^12^	6.7 × 10^11^
*k*_3→1_	3.0 × 10^–25^	2.3 × 10^–23^	7.1 × 10^–26^	1.0 × 10^–25^	9.2 × 10^–25^	3.1 × 10^–24^	1.9 × 10^–25^
*k*_2→3_	1.3 × 10^8^	4.6 × 10^5^	5.1 × 10^3^	1.1 × 10^8^	1.4 × 10^3^	9.6 × 10^3^	5.1 × 10^5^
*k*_3→2_	9.1 × 10^–3^	1.8 × 10^7^	5.4 × 10^1^	7.8 × 10^–3^	2.1 × 10^–5^	2.2 × 10^–4^	3.6 × 10^–5^

a Here, Γ_*jk*_ is the electronic coupling (in meV) between states *j* and *k*, *E*_*r*_^(*jk*)^ is the reorganization energy (in eV) between states *j* and *k*, *ε*_*j*_ is the energy minimum (in eV) of state *j*, and *k*_*j*→*k*_ is the Marcus rate constant (in Hz) for transition *j* → *k*.

**Table 3:** Solvent-Accessible Surface Area (SASA) and Minimal Intermolecular Distances of the Three Conformations (R0, R1, R2) of the MPe/2TCNE Trimer^a^

Conformation	R0	R1	R2
SASA(D)	3.293	3.645	3.604
SASA(A_1_)	1.460	1.989	1.879
SASA(A_2_)	2.158	2.014	2.861
SASA(D, A_1_)	4.753	5.634	5.483
SASA(D, A_2_)	5.451	5.659	6.465
SASA(A_1_, A_2_)	3.618	4.003	4.740
SASA(D, A_1_, A_2_)	6.911	7.648	8.344
*d*(D, A_1_)	2.76	4.56	2.76
*d*(D, A_2_)	2.76	2.76	5.62
*d*(A_1_, A_2_)	3.17	2.75	9.59

a Here, SASA (unitless) is defined for one or multiple solute molecules, including the MPe donor (D) and the two TCNE acceptors (A_1_, A_2_), and *d*(X, Y) is the minimal distance (in Å) between X and Y molecules that can be selected from {D, A_1_, A_2_}.

It is important to note that these Marcus rate constants are utilized here primarily as a diagnostic tool to interpret the dominant kinetic pathways and activation barriers, rather than as quantitative predictions of the full nonadiabatic dynamics, which are rigorously simulated using NAMD approaches. Figure 2 visualizes the Marcus parabolas and the associated rate constants for the three conformations.^49,50^ These rate constants reveal two primary kinetic pathways for the photoinduced CT process:

$$\begin{array}{rll} \left(i\right) & {\mathrm{LE}}_{\left|1\right\rangle }\to \mathrm{CT}1_{\left|2\right\rangle }\to \mathrm{CT}2_{\left|3\right\rangle } & \\ \left(\mathrm{ii}\right) & {\mathrm{LE}}_{\left|1\right\rangle }\to \mathrm{CT}2_{\left|3\right\rangle } \end{array}$$ 12

This identification stems from the observation that only the rate constants *k*_1→2_, *k*_1→3_, and *k*_2→3_ exhibit significant magnitudes.

**Figure 2:**
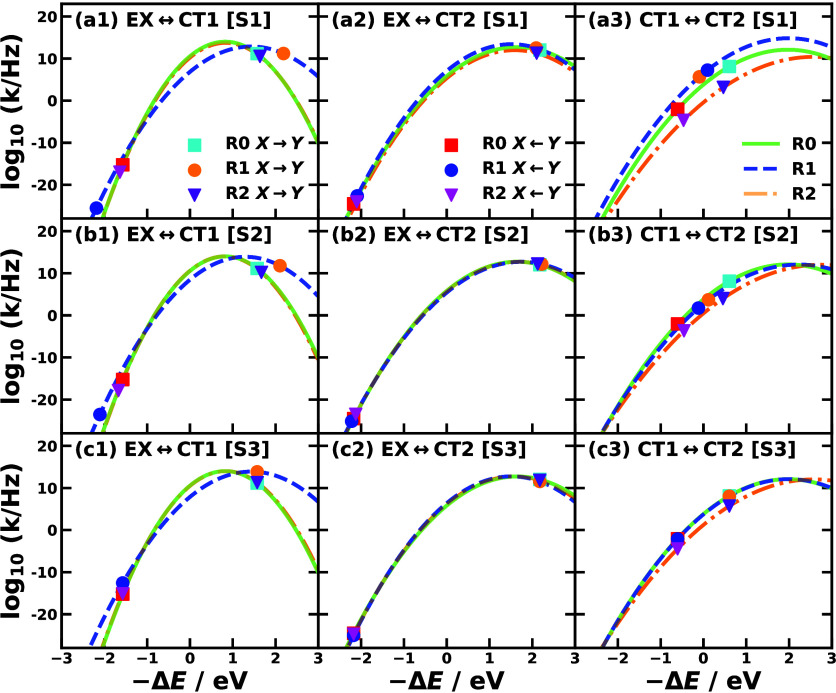
Marcus parabolas and rate constants of MSH models of conformations R0 (green solid and square symbols), R1 (blue dashed and circle symbols), and R2 (yellow dot dashed and triangle symbols) parametrized via Scheme 1 (row a), Scheme 2 (row b), and Scheme 3 (row c). The three columns present the forward reactions (cyan, orange, and purple symbols for R0, R1, and R2) and backward reactions (red, blue, and magenta symbols for R0, R1, and R2) for EX ↔ *CT*1, EX ↔ *CT*2, and CT1 ↔CT2, respectively.

The first question we address is identifying which molecular properties significantly influence the reorganization energies and, consequently, the degree of bath correlation, θ_23_. Tables 1 and 3 suggest that the change in the solute’s permanent dipole moment upon transition plays a primary role, while the solvent-accessible surface area (SASA)^45^ plays a secondary role. For example, comparing Scheme 2 against Scheme 1 for the same conformation (R1 or R2) reveals overestimated reorganization energies for the EX → CT2 (*E*_*r*_^(13)^) and CT1 ↔ CT2 (*E*_*r*_^(23)^) transitions, alongside an underestimated reorganization energy for the EX → CT1 (*E*_*r*_^(12)^) transition. As defined in eq 2, the reorganization energy is determined by the thermal fluctuations of the energy gap between the two PESs, which microscopically corresponds to solvent reorganization upon the electronic transition. Therefore, a more significant rearrangement of solvent molecules results in a larger reorganization energy.

In the context of photoinduced CT within a polar solvent, a substantial change in the electrostatic field induced by the electronic transition of the solute drives a significant rearrangement of the surrounding acetonitrile (ACN) molecules. The magnitude of the solute’s permanent dipole change upon transition, denoted as |Δ**μ**_*XY*_|, serves as the primary metric for characterizing these electrostatic interactions. This metric aligns with the trends in reorganization energy observed between Scheme 1 and Scheme 2 in Table 1. Specifically, |Δ**μ**_12_| decreases, whereas |Δ**μ**_13_| and |Δ**μ**_23_| increase for both the R1 and R2 conformations. We also observe that for a fixed trimer conformation, such as the comparison between R1 [S1] and R1 [S2], a larger mean absolute difference in atomic charges generally corresponds to a larger |Δ**μ**_*XY*_| (see Table S1).

Furthermore, when the set of atomic charges is held constant and only the solute conformation varies, the dipole change upon transition remains strongly correlated with the reorganization energy trends. For instance, the transition from R0 to R1 [S2] exhibits a significantly increased |Δ**μ**_12_|, which implies an increase in *E*_*r*_^(12)^. This shift is directly attributable to the displacement of the TCNE A_1_ molecule 3 Å further away from the MPe donor. However, distinct behaviors emerge when the dipole change is identical. Both R0 and R1 [S2] possess the same |Δ**μ**_13_| values, yet the reorganization energy *E*_*r*_^(13)^ is larger in R1 [S2]. In such cases, the SASA serves as a secondary descriptor where a larger SASA generally leads to a larger reorganization energy. Since the transition EX →CT2 involves the MPe donor and the TCNE A_2_ acceptor, the observed increase in SASA­(D, A_2_) from R0 to R1 [S2] in Table 3 aligns with the corresponding trend in reorganization energy.

Figure 3 shows the photoinduced CT dynamics over the first 2 ps for the three trimer conformations, employing both all-atom and MSH model Hamiltonians parametrized via Schemes 1 and 2. The NAMD simulations were conducted at 300 K using the classical mapping model (CMM) with a zero-point-energy parameter of γ = 0.309.^51–53^ The CMM approach, alongside linearized semiclassical (LSC) dynamics^54,55^ and symmetrical quasiclassical (SQC) dynamics,^56,57^ has been established as a robust method for NAMD with mapping Hamiltonian^58,59^ in condensed-phase systems.^60^ The dynamics obtained from the MSH models align well with the all-atom NAMD results, reaffirming the MSH model’s capacity to accurately capture the essential nonadiabatic dynamics of complex molecular systems in condensed phases, consistent with findings in other systems.^31,38^ The Marcus rate constants listed in Table 2 and visualized in Figure 2 provide a kinetic basis for interpreting the NAMD results in Figure 3. For example, comparing R1 [S1] to R0 reveals that CT1 population growth accelerates while CT2 population growth decelerates. This shift can be attributed to the rate constant *k*_2→3_ for the CT1 →CT2 transition, which is 2 orders of magnitude lower in R1 [S1] than in R0 due to a reduced driving force and increased reorganization energy. Thus, the sequential pathway (i) in eq 12 is hindered. Simultaneously, the rate *k*_1→3_ for the EX →CT2 transition increases in R1 [S1] relative to R0, enhancing the direct pathway (ii). In contrast, the population transfer from the initial EX state to the CT states in conformation R2 [S1] is markedly slower than in the other conformations under Scheme 1. This sluggish dynamics arises because all three primary rate constants are low, a consequence of the weak electronic couplings inherent to the bilateral A-D-A arrangement of conformation R2.

**Figure 3:**
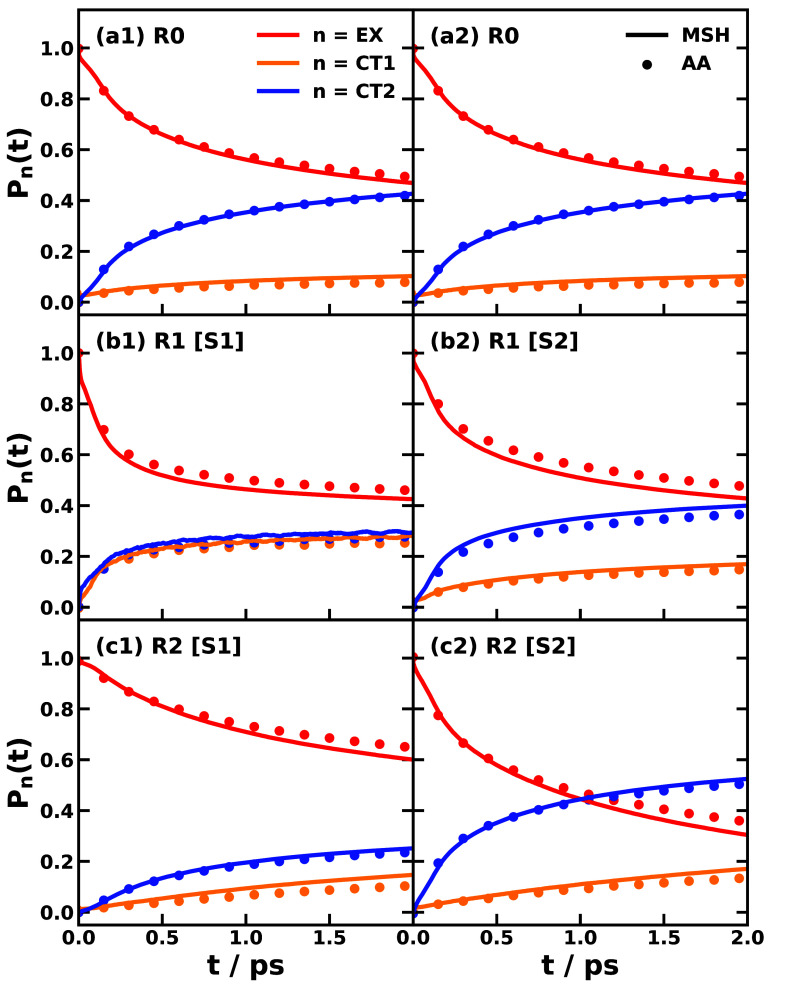
Photoinduced nonadiabatic dynamics obtained with all-atom Hamiltonian (AA, dot) and multistate harmonic model (MSH, solid line) for conformations R0, R1, and R2 of the MPe/2TCNE trimer, where the population of bright EX state (red) transfers to CT1 (orange) and CT2 (blue) states. The nonadiabatic dynamics are simulated via classical mapping model (CMM) with γ = 0.309 at 300 K. Left and right columns correspond to Scheme 1 and Scheme 2, respectively, and panels (a1) and (a2) of R0 are identical.

The second question we address is determining the dynamical signatures associated with different bath correlation scenarios. We compare the photoinduced CT dynamics over the first 10 ps for MSH models constructed using Schemes 1–3 for conformations R1 and R2 against the reference R0 conformation, as plotted in Figure 4.

**Figure 4:**
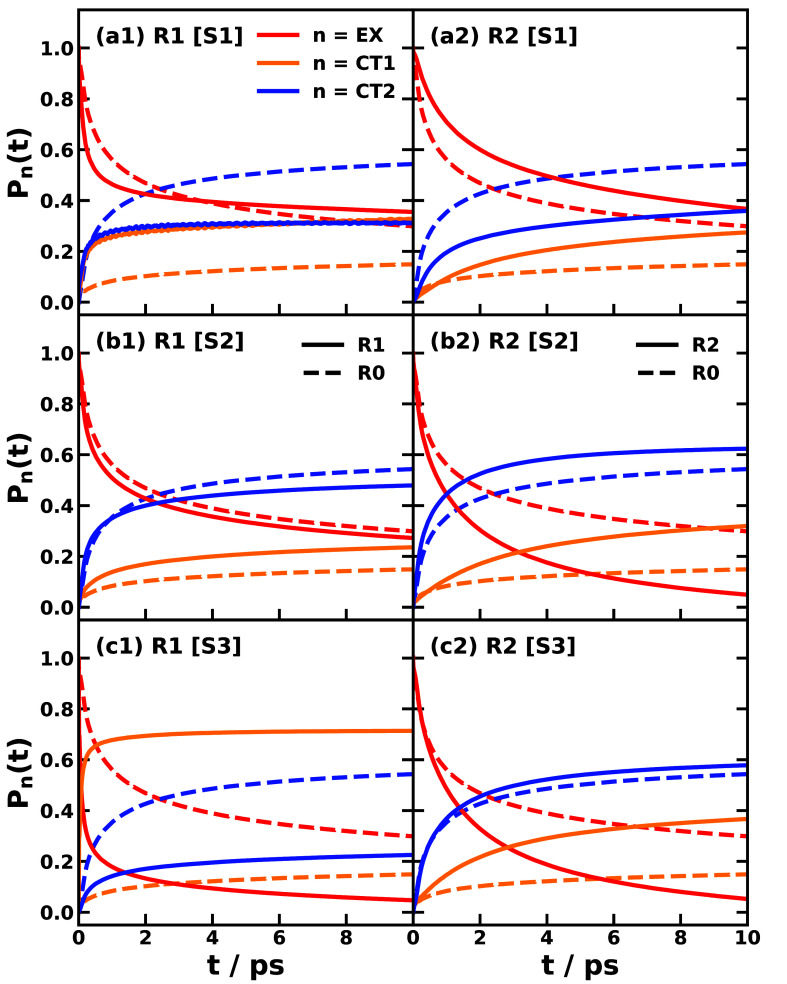
Photoinduced nonadiabatic dynamics obtained with the MSH models (solid line) of Schemes 1–3 (rows a, b, and c) for conformations R1 (left column) and R2 (right column) of the MPe/2TCNE trimer, compared with the MSH model of conformation R0 (dashed line), where the population of bright EX state (red) transfers to CT1 (orange) and CT2 (blue) states. The nonadiabatic dynamics are simulated via CMM with γ = 0.309 at 300 K.

We begin by analyzing the correlated bath case in conformation R1. To isolate the pure bath correlation effect, we compare the dynamics of the reference conformation R0 with that of R1 [S3]. By construction, these two models share an identical system Hamiltonian (electronic couplings and site energies *ε*_*j*_), differing only in their bath parameters. The geometric metric θ_23_ decreases from 77.7° in R0 to 71.0° in R1 [S3], indicating a more strongly correlated bath in the latter. This enhanced bath correlation is intrinsically linked to the increase in the reorganization energy *E*_*r*_^(12)^, which rises from 0.823 eV in R0 to 1.432 eV in R1 [S3]. Physically, this increase stems from the greater donor–acceptor A_1_ separation in R1 geometry, which requires more extensive solvent rearrangement upon charge transfer. The dynamical consequences of this shift are evident in Figure 4(c1): the population decay of the EX state and the corresponding rise of the CT1 population are significantly faster in R1 [S3] than in R0, while the population gain in CT2 is slower. This acceleration of the primary EX →CT1 step is driven by a two-order-of-magnitude increase in the rate constant *k*_1→2_. The enhancement in rate is a direct consequence of the activation energy *E*_act_^(12)^ dropping from 0.17 eV in R0 to a nearly barrierless 0.0034 eV in R1 [S3] (using eq 11), a change ultimately caused by the increased reorganization energy pushing the reaction closer to the activationless regime as shown by cyan square and orange circle in Figure 2(c1).

Moreover, comparing Schemes 2 and 3 allows us to investigate the effects of bath parameters and thermodynamic driving forces. For the transitions of interest, EX →CT1 and EX →CT2, the angle θ_23_ decreases from 74.3° in R1 [S2] to 71.0° in R1 [S3], indicating a stronger bath correlation in the latter. Figure 4(b1,c1) shows that the population decay of the EX state and the growth of the CT1 state proceed more rapidly in R1 [S3] than in R1 [S2]. This acceleration correlates with an increased rate constant *k*_1→2_ in R1 [S3], driven by the fact that the thermodynamic driving force (Δ*E*_12_) almost cancels with the larger reorganization energy (*E*_*r*_^(12)^), which leads to an activation energy *E*_act_^(12)^ of 0.0034 eV in R1 [S3], significantly smaller than 0.213 eV in R1 [S2]. As illustrated by the orange circles in Figure 2(b1,c1), these factors push the reaction to nearly barrierless top of the Marcus turnover in R1 [S3] compared to R1 [S2].

Alternatively, comparing R0 with R1 [S2] isolates the dynamical differences arising solely from the change in solute geometry, as R1 [S2] retains the Hamiltonian parameters of R0. The bath correlation angle θ_23_ shifts from 77.7° in R0 to 74.3° in R1 [S2], suggesting a more highly correlated bath in the modified R1 geometry. Both the short-time dynamics in Figure 3(a2,b2) and the long-time behavior in Figure 4(b1) reveal faster EX decay and CT1 growth in R1 [S2] relative to R0. This behavior is supported by the kinetic data in Table 2 and Figure 2(a1–3,b1–3), which show elevated rate constants *k*_1→2_ and *k*_1→3_, alongside a significantly reduced *k*_2→3_ in R1 [S2]. The enhancement of *k*_1→2_ and *k*_1→3_ results from solvent rearrangement following photoexcitation, which induces larger reorganization energies across the three state pairs, coupled with a larger thermodynamic driving force in the inverted regime in R1 [S2] than R0. Conversely, the suppression of *k*_2→3_ stems primarily from a reduced thermodynamic driving force Δ*E*_23_ of 0.047 eV and an amplified reorganization energy *E*_*r*_^(23)^ of 2.222 eV, leading to a high activation energy of 0.579 eV in R1 [S2] in the normal regime, effectively disrupting the CT1 →CT2 step of pathway (i) defined in eq 12.

Beyond bath parameters and solute geometry, we assess the impact of the system Hamiltonian, specifically electronic couplings and excitation energies, by comparing R1 [S1] and R1 [S3]. Because the TCNE A_1_ molecule is displaced further from the MPe donor in R1, the electronic coupling |Γ_12_| is lower in R1 [S1] compared to the optimized R0 structure used in R1 [S3]. However, |Γ_13_| and |Γ_23_| are higher in R1 [S1] according to Table 2. Figure 4(a1,c1) demonstrates that in R1 [S1], the EX decay and CT1 growth are slower, whereas CT2 growth is faster compared to R1 [S3]. This trend aligns with the kinetic pathway analysis; although the backward reaction CT2 → CT1 is faster than the forward reaction CT1 → CT2, both are orders of magnitude smaller than the suppressed *k*_12_ and enhanced *k*_13_ rates, as shown in Table 2 and Figure 2(a1–3,c1–3).

Next, we examine the anticorrelated bath case represented by conformation R2. To isolate the pure bath correlation effect in the anticorrelated regime, we compare the reference R0 with R2 [S3]. As with the previous comparison, the electronic Hamiltonian remains fixed, so all dynamical differences arise solely from the bath. The correlation metric θ_23_ shifts from 77.7° in R0 to 93.9° in R2 [S3], marking a clear transition to an anticorrelated bath. This regime change is energetically manifested by a significant increase in the reorganization energy *E*_*r*_^(23)^, which rises from 1.934 eV in R0 to 2.444 eV in R2 [S3]. The molecular origin of this increase lies in the geometry of R2: with acceptor A_2_ positioned on the opposite side of the donor relative to A_1_, transitioning between the two CT states requires a massive solvent rearrangement to invert the polarization field across the donor plane. Dynamically, Figure 4(c2) indicates that while the EX decay and CT1 population growth are faster in R2 [S3] than in R0, the accumulation of the CT2 population is similar. This behavior is primarily governed by the suppression of the sequential transfer channel; the rate constant *k*_2→3_ drops by 2 orders of magnitude in R2 [S3] compared to R0. This kinetic blockade is directly caused by the elevated activation energy (rising from 0.228 to 0.346 eV), which is itself a consequence of the large reorganization energy *E*_*r*_^(23)^ characteristic of the anticorrelated bath. The corresponding Marcus parabolas illustrating this barrier increase are shown in Figure 2(c3), comparing cyan squares for R0 and purple triangles for R2 [S3].

To investigate the effects of bath parameters and thermodynamic driving forces, we compare R2 [S3] with R2 [S2], as shown in Figure 4(b2,c2). The degree of bath correlation θ_23_ increases from 93.9° in R2 [S3] to 96.9° in R2 [S2], indicating stronger anticorrelated bath effects in the latter. Figure 4(b2,c2) reveals that while the EX decay rate remains comparable in both schemes, CT1 growth is faster and CT2 growth is slower in R2 [S3] than in R2 [S2]. This behavior can be rationalized by the kinetic data: the rate constant *k*_1→2_ is larger, while *k*_1→3_ is smaller in R2 [S3] compared to R2 [S2], as depicted by the purple triangles in Figure 2(b1,b2,c1,c2). It is noted that *k*_2→3_ is higher in R2 [S3] than in R2 [S2], a consequence of the more evenly distributed charge distribution in R2 [S3] leading to a smaller reorganization energy in the normal regime (Figure 2(b3,c3)).

Alternatively, comparing R0 with R2 [S2] isolates the dynamical impact of the solute geometry change, as both use the same R0 electronic parameters. The bath correlation angle shifts from θ_23_ = 77.7° in R0 (correlated bath) to θ_23_ = 96.9° in R2 [S2] (anticorrelated bath). Both the short-time dynamics in Figure 3(a2,c2) and the long-time behavior in Figure 4(b2) show that EX decay and the growth of both CT1 and CT2 are faster in R2 [S2] than in R0. This acceleration is supported by the fact that the sum of *k*_1→2_ and *k*_1→3_ is higher in R2 [S2], despite *k*_1→2_ and *k*_2→3_ being lower and *k*_1→3_ being higher individually according to Table 2 and Figure 2(a1–3,b1–3). The dramatic four-order-of-magnitude reduction in *k*_2→3_ for R2 [S2] relative to R0 is primarily attributed to a massive increase in the reorganization energy *E*_*r*_^(23)^. This increase reflects the significant solvent rearrangement required when the solute geometry changes from having two acceptors on the same side (R0) to having them on opposite sides of the donor (R2), calling for the accommodation of two widely separated acceptor molecules with flipped charge distributions.

Finally, comparing R2 [S1] with R2 [S3] elucidates the effects of the system Hamiltonian, specifically the electronic couplings and excitation energies. While the thermodynamic driving forces remain similar, the electronic couplings are significantly smaller in R2 [S1], which features the bilateral A-D-A arrangement, compared to R2 [S3], which retains the couplings computed for the R0 geometry (Table 2). Consequently, Figure 4(a2,c2) shows that EX decay and the growth of both CT1 and CT2 are slower in R2 [S1] than in R2 [S3]. This slowdown is expected, as all dominant reaction rates in the two kinetic pathways defined in eq 12 are reduced, as detailed in Table 2 and Figure 2(a1–3,c1–3).

Beyond analyzing population dynamics, we can extract atomistic details from the nonadiabatic simulations of the three trimer conformations. Figure 5 plots the changes in the radial distribution function (RDF) for various solute–solvent components at finite time points (0.5, 1, and 4 ps) relative to time zero (photoexcitation).^31,61^ These time slices were derived directly from the all-atom nonadiabatic simulation of conformation R1, conducted using SQC dynamics with a triangle window function.^56,57^ In the first row of Figure 5, we observe a general tendency for the entire solute trimer to slightly repel ACN solvent molecules within a 3–5 Å range from the molecular surface. Specifically, the MPe donor repels ACN molecules, whereas the two TCNE acceptors attract the polar solvent, inducing a shell-like rearrangement at distances of approximately 2–7 Å. The second and third rows of Figure 5 decompose these interactions by displaying the solute moieties relative to the negative N atom and positive H atoms of ACN, respectively. It is evident that as the MPe donor becomes positively charged during the NAMD, it attracts the ACN nitrogen atoms and repels the hydrogen atoms (Figure 5(b2,c2)). Conversely, the two TCNE acceptors (A_1_, A_2_), which gradually acquire negative charge, exhibit the opposite behavior (Figure 5(b3,b4,c3,c4)). We propose a semiquantitative relationship for the RDF changes, positing that the intensity of the TCNE acceptors’ interaction with the solvent atoms correlates directly with the product of the CT1 or CT2 population at a given time and the SASA of the corresponding acceptor. For instance, in conformation R1 at 4 ps, the populations of both CT1 and CT2 are approximately 0.1, and the SASA values for both TCNE A_1_ and A_2_ are roughly 0.7. Consequently, we anticipate the dominant peak height ratio A_1_:A_2_ to be near 1:1, a prediction confirmed by the data in Figure 5(b3,b4) and (c3,c4). Additional RDF data for the equilibrated ground state (time 0) of all conformations, as well as the RDF evolution for other conformations, are provided in the Supporting Information.

**Figure 5:**
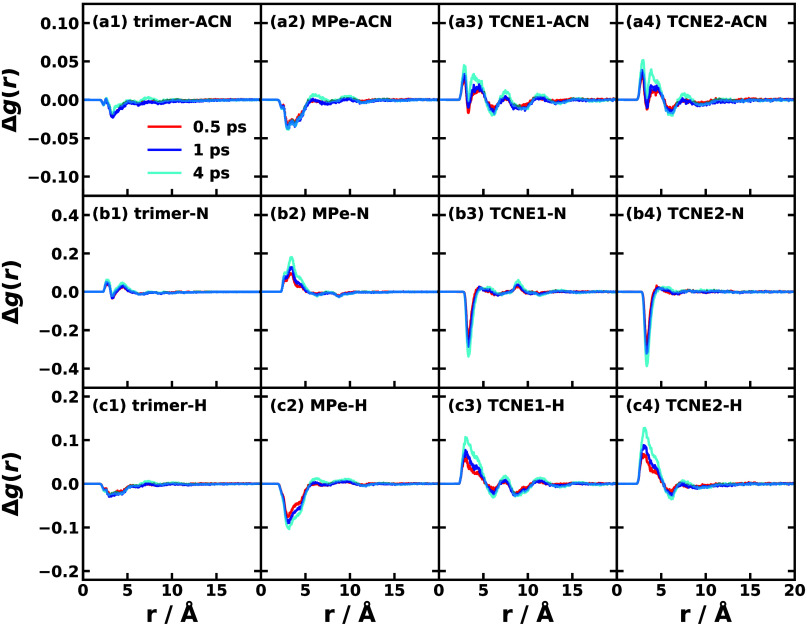
Nonequilibrium solute–solvent radial distribution function (RDF) evolution Δ*g*(*r*) of conformation R1 of MPe/2TCNE trimer at times of *t* = 0.5 (red), 1 (blue), and 4 ps (cyan) with respect to the initial photoexcitation time *t* = 0. The four columns correspond to the surface of the whole trimer (column 1), MPe donor (column 2), TCNE No. 1 (column 3), and TCNE No. 2 (column 4) solute with respect to the different solvent parts, respectively; the three rows correspond to all ACN solvent atoms (row a), the N atoms of ACN (row b), and the H atoms of ACN (row c), respectively. The nonadiabatic dynamics are simulated with the all-atom Hamiltonian (Scheme 1) using SQC dynamics with the triangle window at 300 K.

As discussed above, nonadiabatic dynamics of photoinduced CT processes in polar solvents are influenced by multiple factors, including electronic couplings, excitation energies, bath parameters, and system-bath couplings. However, the primary objective of this work is to establish a molecular perspective of bath correlation, regardless of electronic couplings and vertical excitation energies. In particular, we extract the fundamental distinctions between correlated and anticorrelated baths from our findings. First, the degree of bath correlation is quantified by the angle θ_23_ between the two transitions of interest, |1⟩→|2⟩ and |1⟩→|3⟩, which is directly determined by the three pairwise reorganization energies according to eq 1. Physically, the reorganization energy reflects the fluctuations of the energy gap between corresponding states, which are governed by both molecular conformation and charge distribution. In the present case, polar solvent molecules respond to changes in the solute’s conformation and charge distribution upon electronic transition, making the solvent’s rearrangement a major contributor to the reorganization energy. On the molecular level, molecular conformation and charge distribution determine the dipole moment change, and molecular conformation determines the SASA of the solute, so the dipole change and SASA serve as a molecular indicator for the reorganization energy.

The physical picture of a correlated bath for the |1⟩→|2⟩ and |1⟩→|3⟩ reactions is that in-phase bath fluctuations drive the concerted progression of both reactions. This regime is characterized by a relatively small *E*_*r*_^(23)^, indicating low energy-gap fluctuations between states |2⟩ and |3⟩. Within the MSH model or the equivalent multistate reaction coordinate (MRC) model, this relationship corresponds to a short equilibrium distance between these states along the collective reaction coordinate. Thus, in a correlated bath, a change in molecular conformation that increases *E*_*r*_^(12)^ is expected to simultaneously increase *E*_*r*_^(13)^. Generalizing this principle, we anticipate that correlated baths will be expected in cascade-type architectures (e.g., D–A_1_–A_2_ arrays) where sequential charge transfer steps involve charge displacements in similar spatial directions; here, the solvent polarization induced by the first step naturally preorganizes the environment for the second.

In contrast, the picture of an anticorrelated bath describes a scenario where a bath fluctuation favoring one reaction, such as |1⟩→|2⟩, disfavors the alternative reaction, |1⟩→|3⟩. This behavior resembles out-of-phase fluctuations and is characterized by a relatively large *E*_*r*_^(23)^. It can be understood as a situation where the two reactions require solvent motions in opposing directions, reflected by a large separation between the equilibrium positions of states |2⟩ and |3⟩ along the collective reaction coordinate. Accordingly, such anticorrelated baths are likely to be common in branched or highly symmetric geometries (such as the sandwich conformation R2 studied here) where alternative pathways involve spatially opposing charge transfer patterns. In these scenarios, the solvent shell cannot simultaneously stabilize both distinct charge-transfer states, leading to the competitive frustration characteristic of the anticorrelated regime.

These theoretical distinctions of bath regimes may translate into specific, measurable experimental signatures, such as kinetic branching ratios and spectroscopic lineshapes. Kinetically, our simulations predict that an anticorrelated bath creates a dynamical bottleneck by blocking the sequential pathway; experimentally, this would be observable as a distinct alteration in the kinetic branching ratio, manifesting as the transient accumulation of the intermediate species and a suppressed quantum yield of the final product state, in contrast to the efficient sequential transfer typical of correlated-bath cascade geometries. Spectroscopically, two-dimensional electronic spectroscopy (2DES) offers a direct probe of these correlations,^62^ as the geometric angle θ_23_ maps to the cross-correlation of energy gap fluctuations. Consequently, we predict that the correlated bath regime will be signaled by cross-peaks with a substantial positive center line slope, indicating synchronous stabilization of the EX and CT manifolds, whereas the anticorrelated regime will exhibit cross-peaks with a negative center line slope, reflecting the opposing energetic demands of the competing pathways.

In conclusion, we establish a uniquely defined metric for bath correlation between two nonadiabatic transitions sharing an initial state, |1⟩→|2⟩ and |1⟩→|3⟩. This metric is the angle θ_23_, which is fully determined by the three reorganization energies *E*_*r*_^(12)^, *E*_*r*_^(13)^, and *E*_*r*_^(23)^ via eq 1. An angle θ_23_ less than 90° indicates a correlated bath, whereas an angle θ_23_ greater than 90° indicates an anticorrelated bath. These reorganization energies are intrinsically linked to the molecular properties of the electronic states and are strongly affected by changes in dipole moment and SASA upon electronic transition. Thus, the specific molecular conformation and charge distribution determine whether a molecule or aggregate in a polar solvent falls within the correlated or anticorrelated bath regime. Within a correlated bath, energy-gap fluctuations and solvent motions facilitate both reactions, associated with a small *E*_*r*_^(23)^. Conversely, within an anticorrelated bath, solvent motions favor one reaction at the expense of the other, associated with a large *E*_*r*_^(23)^. This study offers a clear physical picture of the correlated bath effect for nonadiabatic dynamics in polar solvents, while the investigation of bath effects on more complex dynamics in disordered heterogeneous environments^63–66^ will be reported in future work.

## Supplementary Material



## Notes

Simulation details of nonadiabatic dynamics, MSH schemes and triangles, NAMD obtained with mean-field Ehrenfest, SQC with triangle windows, LSC mapping dynamics, as well as the equilibrium ground-state RDFs and nonequilibrium RDFs for the three conformations of the MPe/2TCNE trimer in acetonitrile (PDF)

## References

[1] Mahadevan S., Liu T., Pratik S. M., Li Y., Ho H. Y., Ouyang S., Lu X., Yip H.-L., Chow P. C. Y., Brédas J.-L. (2024). Assessing Intra- and Inter-Molecular Charge Transfer Excitations
in Non-Fullerene Acceptors Using Electroabsorption Spectroscopy. Nat. Commun..

[2] Guo J., Qin S., Zhang J., Zhu C., Xia X., Gong Y., Liang T., Zeng Y., Han G., Zhuo H. (2025). Asymmetric Small-Molecule Acceptor Enables
Suppressed Electron-Vibration
Coupling and Minimized Driving Force for Organic Solar Cells. Nat. Commun..

[3] Li C., Song J., Lai H., Zhang H., Zhou R., Xu J., Huang H., Liu L., Gao J., Li Y. (2025). Non-Fullerene Acceptors
with High Crystallinity and Photoluminescence
Quantum Yield Enable > 20% Efficiency Organic Solar Cells. Nat. Mater..

[4] Stojanovic L., Giannini S., Blumberger J. (2024). Exciton Transport
in the Nonfullerene
Acceptor O-IDTBR from Nonadiabatic Molecular Dynamics. J. Chem. Theory Comput..

[5] Navickas T., MacDonell R. J., Valahu C. H., Olaya-Agudelo V. C., Scuccimarra F., Millican M. J., Matsos V. G., Nourse H. L., Rao A. D., Biercuk M. J. (2025). Experimental Quantum
Simulation of Chemical Dynamics. J. Am. Chem.
Soc..

[6] Ma X., Tian X., Stippell E., Prezhdo O. V., Long R., Fang W.-H. (2024). Self-passivation
of Halide Interstitial Defects by
Organic Cations in Hybrid Lead-Halide Perovskites: Ab Initio Quantum
Dynamics. J. Am. Chem. Soc..

[7] Costa G. J., Liang R. (2025). Decrypting the Nonadiabatic
Photoinduced Electron Transfer Mechanism
in Light-Sensing Cryptochrome. ACS Cent. Sci..

[8] Cho K. H., Jang S. J., Rhee Y. M. (2024). Dynamic
Embedding of Effective Harmonic
Normal Mode Vibrations in All-Atomistic Energy Gap Fluctuations: Case
Study of Light Harvesting 2 Complex. J. Chem.
Phys..

[9] Romero E., Novoderezhkin V. I., van Grondelle R. (2017). Quantum Design of Photosynthesis
for Bio-Inspired Solar-Energy Conversion. Nature.

[10] Nelson T. R., White A. J., Bjorgaard J. A., Sifain A. E., Zhang Y., Nebgen B., Fernandez-Alberti S., Mozyrsky D., Roitberg A. E., Tretiak S. (2020). Non-Adiabatic Excited-State
Molecular Dynamics: Theory
and Applications for Modeling Photophysics in Extended Molecular Materials. Chem. Rev..

[11] Crespo-Otero R., Barbatti M. (2018). Recent Advances and Perspectives
on Nonadiabatic Mixed
Quantum–Classical Dynamics. Chem. Rev..

[12] Liu X.-Y., Chen W.-K., Fang W.-H., Cui G. (2023). Nonadiabatic Dynamics
Simulations for Photoinduced Processes in Molecules and Semiconductors:
Methodologies and Applications. J. Chem. Theory
Comput..

[13] Nitzan, A. Chemical Dynamics in Condensed Phases: Relaxation, Transfer and Reactions in Condensed Molecular Systems; Oxford University Press: 2006.

[14] May, V. ; Kühn, O. Charge and Energy Transfer Dynamics in Molecular Systems; Wiley: 2011.

[15] Weiss, U. Quantum Dissipative Systems; World Scientific: 2012.

[16] Xu D., Schulten K. (1994). Coupling of Protein
Motion to Electron Transfer in
a Photosynthetic Reaction Center: Investigating the Low Temperature
Behavior in the Framework of the Spin-Boson Model. Chem. Phys..

[17] Lambert N., Ahmed S., Cirio M., Nori F. (2019). Modelling
the Ultra-strongly
Coupled Spin-Boson Model with Unphysical Modes. Nat. Commun..

[18] Caldeira A. O., Leggett A. J. (1983). Quantum Tunnelling
in a Dissipative System. Ann. Phys..

[19] Makri N. (1999). The Linear
Response Approximation and Its Lowest Order Corrections: An Influence
Functional Approach. J. Phys. Chem. B.

[20] Adolphs J., Renger T. (2006). How Proteins Trigger
Excitation Energy Transfer in
the FMO Complex of Green Sulfur Bacteria. Biophys.
J..

[21] Ishizaki A., Fleming G. R. (2012). Quantum Coherence in Photosynthetic Light Harvesting. Annu. Rev. Condens. Matter Phys..

[22] Silbey R. (1976). Electronic
Energy Transfer in Molecular Crystals. Annu.
Rev. Phys. Chem..

[23] Cho M., Silbey R. J. (1995). Nonequilibrium Photoinduced
Electron Transfer. J. Chem. Phys..

[24] Strümpfer J., Schulten K. (2011). The Effect of Correlated
Bath Fluctuations on Exciton
Transfer. J. Chem. Phys..

[25] Popp W., Polkehn M., Hughes K. H., Martinazzo R., Burghardt I. (2019). Vibronic Coupling Models for Donor-Acceptor
Aggregates
Using an Effective-Mode Scheme: Application To Mixed Frenkel And Charge-Transfer
Excitons in Oligothiophene Aggregates. J. Chem.
Phys..

[26] Jang S. J., Mennucci B. (2018). Delocalized Excitons
in Natural Light-Harvesting Complexes. Rev.
Mod. Phys..

[27] Makri N. (2023). Topological
Aspects of System-Bath Hamiltonians and a Vector Model for Multisite
Systems Coupled to Local, Correlated, or Common Baths. J. Chem. Phys..

[28] Brey D., Burghardt I. (2024). Coherent Transient Localization Mechanism of Interchain
Exciton Transport in Regioregular P3HT: A Quantum-Dynamical Study. J. Phys. Chem. Lett..

[29] Yang L., Jang S. J. (2020). Theoretical Investigation of Non-Förster
Exciton
Transfer Mechanisms in Perylene Diimide Donor, Phenylene Bridge, and
Terrylene Diimide Acceptor Systems. J. Chem.
Phys..

[30] Lee C. K., Shi L., Willard A. P. (2019). Modeling
the Influence of Correlated Molecular Disorder
on the Dynamics of Excitons in Organic Molecular Semiconductors. J. Phys. Chem. C.

[31] Hu Z., Sun X. (2022). All-Atom Nonadiabatic Semiclassical Mapping Dynamics
for Photoinduced
Charge Transfer of Organic Photovoltaic Molecules in Explicit Solvents. J. Chem. Theory Comput..

[32] Stratt R. M., Adams J. E. (1993). Solvation by Nonpolar Solvents: Shifts
of Solute Electronic
Spectra. J. Chem. Phys..

[33] Stratt R. M. (1995). The Instantaneous
Normal-Modes of Liquids. Acc. Chem. Res..

[34] Stratt R. M., Maroncelli M. (1996). Nonreactive
Dynamics in Solution: The Emerging Molecular
View of Solvation Dynamics and Vibrational Relaxation. J. Phys. Chem..

[35] Ladanyi B. M., Stratt R. M. (1995). Short-Time Dynamics of Solvation: Linear Solvation
Theory for Polar Solvents. J. Phys. Chem..

[36] Dutta R., Bagchi K., Bagchi B. (2017). Role of Quantum
Coherence in Shaping
the Line Shape of an Exciton Interacting with a Spatially and Temporally
Correlated Bath. J. Chem. Phys..

[37] Olbrich C., Kleinekathöfer U. (2010). Time-Dependent
Atomistic View on
the Electronic Relaxation in Light-Harvesting System II. J. Phys. Chem. B.

[38] Liu Z., Zeng H., Sun X. (2025). Consistent and Generalizable Effective
Model Hamiltonian Framework for Studying Nonadiabatic Dynamics in
the Condensed Phase. J. Chem. Theory Comput..

[39] Hu Z., Brian D., Sun X. (2021). Multi-State
Harmonic Models with
Globally Shared Bath for Nonadiabatic Dynamics in the Condensed Phase. J. Chem. Phys..

[40] Dereka B., Koch M., Vauthey E. (2017). Looking at Photoinduced Charge Transfer
Processes in the IR: Answers to Several Long-Standing Questions. Acc. Chem. Res..

[41] Mohammed O. F., Vauthey E. (2008). Simultaneous Generation of Different
Types of Ion Pairs
upon Charge-Transfer Excitation of a Donor-Acceptor Complex Revealed
by Ultrafast Transient Absorption Spectroscopy. J. Phys. Chem. A.

[42] Mohammed O. F., Adamczyk K., Banerji N., Dreyer J., Lang B., Nibbering E. T. J., Vauthey E. (2008). Direct Femtosecond Observation of
Tight and Loose Ion Pairs upon Photoinduced Bimolecular Electron Transfer. Angew. Chem., Int. Ed..

[43] Koch M., Rosspeintner A., Adamczyk K., Lang B., Dreyer J., Nibbering E. T. J., Vauthey E. (2013). Real-Time Observation of the Formation
of Excited Radical Ions in Bimolecular Photoinduced Charge Separation:
Absence of the Marcus Inverted Region Explained. J. Am. Chem. Soc..

[44] Sun X., Zhang P., Lai Y., Williams K. L., Cheung M. S., Dunietz B. D., Geva E. (2018). Computational Study of Charge-Transfer
Dynamics in the Carotenoid–Porphyrin–C_60_ Molecular
Triad Solvated in Explicit Tetrahydrofuran and Its Spectroscopic Signature. J. Phys. Chem. C.

[45] Shrake A., Rupley J. (1973). Environment and Exposure to Solvent
of Protein Atoms. Lysozyme and Insulin. J. Mol.
Biol..

[46] Marcus R. A. (1956). On the
Theory of Oxidation–Reduction Reactions Involving Electron
Transfer. I. J. Chem. Phys..

[47] Marcus R. A. (1956). Electrostatic
Free Energy and Other Properties of States Having Nonequilibrium Polarization. I. J. Chem. Phys..

[48] Marcus R., Sutin N. (1985). Electron Transfers in Chemistry and Biology. Biochim. Biophys. Acta, Rev. Bioenerg..

[49] Tong Z., Gao X., Cheung M. S., Dunietz B. D., Geva E., Sun X. (2020). Charge Transfer
Rate Constants for the Carotenoid–Porphyrin–C_60_ Molecular Triad Dissolved in Tetrahydrofuran: The Spin-Boson Model
vs the Linearized Semiclassical Approximation. J. Chem. Phys..

[50] Liu Z., Brian D., Sun X. (2024). PyCTRAMER: A Python Package for Charge
Transfer Rate Constant of Condensed-Phase Systems from Marcus Theory
to Fermi’s Golden Rule. J. Chem. Phys..

[51] Liu J. (2016). A Unified
Theoretical Framework for Mapping Models for the Multi-State Hamiltonian. J. Chem. Phys..

[52] He X., Liu J. (2019). A New Perspective for Nonadiabatic Dynamics with Phase Space Mapping
Models. J. Chem. Phys..

[53] He X., Gong Z., Wu B., Liu J. (2021). Negative Zero-Point-Energy
Parameter in the Meyer-Miller Mapping Model for Nonadiabatic Dynamics. J. Phys. Chem. Lett..

[54] Saller M. A. C., Kelly A., Richardson J. O. (2019). On the
Identity of the Identity Operator
in Nonadiabatic Linearized Semiclassical Dynamics. J. Chem. Phys..

[55] Gao X., Saller M. A. C., Liu Y., Kelly A., Richardson J. O., Geva E. (2020). Benchmarking Quasiclassical Mapping Hamiltonian Methods for Simulating
Electronically Nonadiabatic Molecular Dynamics. J. Chem. Theory Comput..

[56] Cotton S. J., Miller W. H. (2013). Symmetrical Windowing for Quantum
States in Quasi-Classical
Trajectory Simulations: Application to Electronically Non-Adiabatic
Processes. J. Chem. Phys..

[57] Cotton S. J., Miller W. H. (2016). A New Symmetrical
Quasi-Classical Model for Electronically
Non-Adiabatic Processes: Application to the Case of Weak Non-Adiabatic
Coupling. J. Chem. Phys..

[58] Meyer H.-D., Miller W. H. (1979). A Classical Analog
for Electronic Degrees of Freedom
in Nonadiabatic Collision Processes. J. Chem.
Phys..

[59] Stock G., Thoss M. (1997). Semiclassical Description
of Nonadiabatic Quantum Dynamics. Phys. Rev.
Lett..

[60] Liu Z., Lyu N., Hu Z., Zeng H., Batista V. S., Sun X. (2024). Benchmarking
Various Nonadiabatic Semiclassical Mapping Dynamics Methods with Tensor-Train
Thermo-Field Dynamics. J. Chem. Phys..

[61] Hu Z., Tong Z., Cheung M. S., Dunietz B. D., Geva E., Sun X. (2020). Photoinduced Charge
Transfer Dynamics in the Carotenoid–Porphyrin–C_60_ Triad via the Linearized Semiclassical Nonequilibrium Fermi’s
Golden Rule. J. Phys. Chem. B.

[62] Schlau-Cohen G. S., Ishizaki A., Fleming G. R. (2011). Two-Dimensional
Electronic Spectroscopy
and Photosynthesis: Fundamentals and Applications to Photosynthetic
Light-Harvesting. Chem. Phys..

[63] Chaudhuri S., Hedström S., Méndez-Hernández D. D., Hendrickson H. P., Jung K. A., Ho J., Batista V. S. (2017). Electron
Transfer Assisted by Vibronic Coupling from Multiple Modes. J. Chem. Theory Comput..

[64] Winte K., Souri S., Lünemann D. C., Zheng F., Madjet M. E.-A., Frauenheim T., Kraus T., Mena-Osteritz E., Bäuerle P., Tretiak S. (2025). Vibronic Coupling-Driven Symmetry
Breaking and Solvation in the Photoexcited Dynamics of Quadrupolar
Dyes. Nat. Chem..

[65] Wang R., Zhang C., Li Q., Zhang Z., Wang X., Xiao M. (2020). Charge Separation from an Intra-Moiety
Intermediate State in the
High-Performance PM6:Y6 Organic Photovoltaic Blend. J. Am. Chem. Soc..

[66] Oberg C. P., Spangler L. C., Coker D. F., Scholes G. D. (2024). Chirped Laser Pulse
Control of Vibronic Wavepackets and Energy Transfer in Phycocyanin
645. J. Phys. Chem. Lett..

