# Supplementary material for: Molecular Origin of Correlated Bath Effects in Photoinduced Charge Transfer Dynamics in Polar Solvents
Source: J Phys Chem Lett. 2026 Mar 16;17(12):3406–18. doi: 10.1021/acs.jpclett.5c04090 (PMC13034464; doi:10.1021/acs.jpclett.5c04090)
Supplement: Supplementary file 1 [file jz5c04090_si_001.pdf]

Supporting Information:
Molecular Origin of Correlated Bath Effects in Photoinduced Charge Transfer Dynamics in Polar Solvents

Xiang Sun[1, 2, 3, 4, 5, a)] and Zengkui Liu[1, 2, 3, 4]
[1)]*Division of Arts and Sciences, NYU Shanghai, 567 West Yangsi Road, Shanghai 200124, China*
[2)]*Department of Chemistry, New York University, New York, New York 10003, United States*
[3)]*NYU-ECNU Center for Computational Chemistry at NYU Shanghai, 3663 Zhongshan Road North, Shanghai 200062, China*
[4)]*Center for Data Science, NYU Shanghai, 567 West Yangsi Road, Shanghai, 200124, China*
[5)]*State Key Laboratory of Precision Spectroscopy, East China Normal University, Shanghai 200062, China*

I. SIMULATION DETAILS OF MPE/2TCNE TRIMER

A. Electronic Structure Calculation

The trimer structure R0 is optimized using the PM6 Hamiltonian using Gaussian16.[1,2] We performed time-dependent density functional theory (TDDFT) under Tamm-Dancoff approximation (TDA) for R0, R1, and R2 conformations at the level of PBE0/6-31+G(d) to obtain excitation energies, electronic couplings using fragment charge difference (FCD) approach,[3] and Merz-Kollman atomic partial charges using Q-Chem 6.[4–7]

B. Molecular Dynamics Simulation

The multistate force fields are obtained by changing state-dependent atomic partial charges into the general AMBER force field (FF).[8] Here, charges are obtained from the quantum chemistry calculation. The simulated system contains one MPe/2TCNE trimer and 1440 acetonitrile solvents. The potential energies of the state j is

$$V_j(\mathbf{R}) = V_j^{\mathrm{FF}}(\mathbf{R}) + W_j(\mathbf{r}^{\mathrm{tri}}), \tag{S1}$$

where $\mathbf{R}$ is all nuclear configurations and $\mathbf{r}^{\mathrm{tri}}$ denotes nuclear configurations of the solute trimer. The state-dependent correction adjusts the force field energies of the gas-phase solute by

$$W_j(\mathbf{r}^{\mathrm{tri}}) = E_j(\mathbf{R}) - V_j^{\mathrm{FF}}(\mathbf{r}^{\mathrm{tri}}), \tag{S2}$$

where E_j is the gas-phase excitation energy and V_j^{FF} denotes the gas-phase modified GAFF excitation energies. State index $j = \{1,2,3,4\}$ corresponds to the brightest state (EX), charge transfer states #1 (CT1) and #2 (CT2), and the ground state (GS), respectively.

All molecular dynamics are performed using the QCDyn package equipped with a modified OpenMM 7.5.0.[9] The nuclear variables are integrated using a time step of $\delta t = 1$ fs. Electrostatic interactions are evaluated using particle mesh Ewald (PME).[10] The PME interaction is truncated in the real space at 9 Å along with the van der Waals interaction. All hydrogen covalent bonds are constrained using the SHAKE algorithm.[11] The solute trimer is restrained using a harmonic force with the Hooke constant of 100 kcal mol^{-1}Å^{-2}. Langevin thermostat is used in NVT and NPT simulations with a friction coefficient of 1 ps^{-1}. The Monte-Carlo barostat is used every 25 steps in the NPT simulation.

The MD simulation for multistate harmonic (MSH) model parameterization follows the listed steps with the EX state force field.

1. Energy minimization: Simulation stops when the iteration energy difference hits the tolerance of 10 kJ mol^{-1} or 10^4 cycles of minimization are performed.

[a)]Electronic mail: xiang.sun@nyu.edu

2. Heating: The box is warmed up from 0 K to 300 K, gradually, during 100 ps using the Langevin thermostat.

3. NPT Equilibration: The box was equilibrated at 300 K and 1 bar to reach the box density at 0.78 g cm^{-3}.

4. NVT Equilibration: NVT simulation of the box lasts 2 ns.

5. NVT Sampling: Initial conditions of NVE sampling are sampled from NVT trajectories with an interval of 100 ps for the following NVE dynamics and an interval of 1 ps for nonadiabatic molecular dynamics.

6. NVE Dynamics: After an NVE relaxation lasting 50 ps, the equilibrated NVE trajectories of 100 ps are utilized to calculate the energies of all four states of interest.

The MSH models are calculated from the energy gap time correlation functions (TCF) of all possible transitions, and 10^7 snapshots are averaged for TCFs.

The MD simulation for MSH parameterization is slightly different from the MD sampling for nonadiabatic molecular dynamics: from the NVT equilibration step, the dynamics are propagated on the bright EX state.

C. Nonadiabatic Molecular Dynamics

The nonadiabatic molecular dynamics are propagated using various semiclassical mapping dynamics at 300 K using the QCDyn package. The initial nuclear positions and velocities are sampled with the MD simulation following the first 5 steps of the MSH parameterization, but changing the propagation state to the ground state. The all-atom nonadiabatic dynamical results via classical mapping model (CMM) dynamics are averaged over 10^5 trajectories with the propagation step length of 0.5 fs using the leapfrog algorithm for nuclear degrees of freedom and the eighth-order Runge-Kutta (RK8) algorithm for electronic degrees of freedom. The all-atom nuclear dynamics via symmetrical quasiclassical (SQC) with triangle windowing function dynamics are averaged over 4×10^4 trajectories with the propagation step length of 0.5 fs using the leapfrog algorithm for nuclear degrees of freedom and the eighth-order Runge-Kutta (RK8) algorithm for electronic degrees of freedom. The MSH results are averaged over 10^6 trajectories with a nuclear propagation step length of 1 fs using velocity-Verlet and an electronic step length of 0.05 fs using the RK4 algorithm.

II. SUPPLEMENTARY TABLES AND FIGURES

The atomic partial charges of the jth electroic state is $\mathbf{C}_j = \{C_i^{(j)} | i = 1, \cdots, N_{\text{atom}}\}$, and N_{atom} is the atomic number of the trimer solute. Differences of atomic partial charges due to the transition $X \to Y$ are $\Delta\mathbf{C}_{XY} = \{\Delta C_i^{(XY)} | i = 1, \cdots, N_{\text{atom}}\}$, where $\Delta C_i^{(XY)} = C_i^{(X)} - C_i^{(Y)}$ is the charge difference between states X and Y on the ith atom. In Table S1, mean absolute differences of atomic partial charge differences for the transition $X \to Y$ are

$$\mathrm{MAD}(\Delta\mathbf{C}_{\mathrm{XY}}) = \frac{1}{\mathrm{N}_{\mathrm{atom}}} \sum_{\mathrm{i}=1}^{\mathrm{N}_{\mathrm{atom}}} \left| \Delta\mathrm{C}_{\mathrm{i}}^{(\mathrm{XY})} \right|. \tag{S3}$$

TABLE S1. Mean absolute difference (MAD) of atomic partial charges of the three conformations (R0, R1, R2) of MPe/2TCNE trimer obtained from Scheme 1 [S1] and Scheme 2 [S2] for different pairs of transitions defined in Eq. S3.

Transitions	R0	R1 [S1]	R1 [S2]	R2 [S1]	R2 [S2]
MAD($\Delta\mathbf{C}_{12}$)	0.046	0.046	0.046	0.048	0.046
MAD($\Delta\mathbf{C}_{13}$)	0.079	0.050	0.079	0.061	0.079
MAD($\Delta\mathbf{C}_{23}$)	0.061	0.049	0.061	0.059	0.061

TABLE S2. Summary of the MSH model parameterization schemes used for the target conformations R1 and R2 with reference conformation R0. "Target" refers to parameters derived directly from the target geometry (R1 or R2), while "Reference" refers to parameters taken from the R0 conformation. "Mixed" indicates that the parameters are recalculated for the target geometry but using the excitation energy and force field of the R0 conformation.

**Scheme**	**Scheme 1 [S1]** *(Fully Consistent)*	**Scheme 2 [S2]** *(Geometry Effect)*	**Scheme 3 [S3]** *(Bath Effect)*
**Objective**	Captures the realistic nonadiabatic dynamics for specific geometry, including consistent electronic and bath parameters.	Isolates solute geometry effect, while keeping electronic parameters and force fields identical to R0.	Isolates pure bath effect by mixing the system Hamiltonian of R0 and the bath parameters of S1.
**Electronic Coupling** (Γ_{jk})	**Target** (R1/R2) *Calculated from target simulations*	**Reference** (R0) *Fixed to R0 values*	**Reference** (R0) *Fixed to R0 values*
**Driving Force** (ΔE, ε_j)	**Target** (R1/R2) *Calculated from target simulations*	**Mixed** *Excitation energy fixed to R0, but* ε_j *are recalculated with target simulations using R0 force field*	**Reference** (R0) *Fixed to R0 values*
**Bath Parameters** (ω_i, $S_i^{(aj)}$, E_r)	**Target** (R1/R2) *Calculated from target simulations*	**Mixed** *Recalculated from target simulations using R0 force field*	**Target** (R1/R2) *Calculated from target simulations*

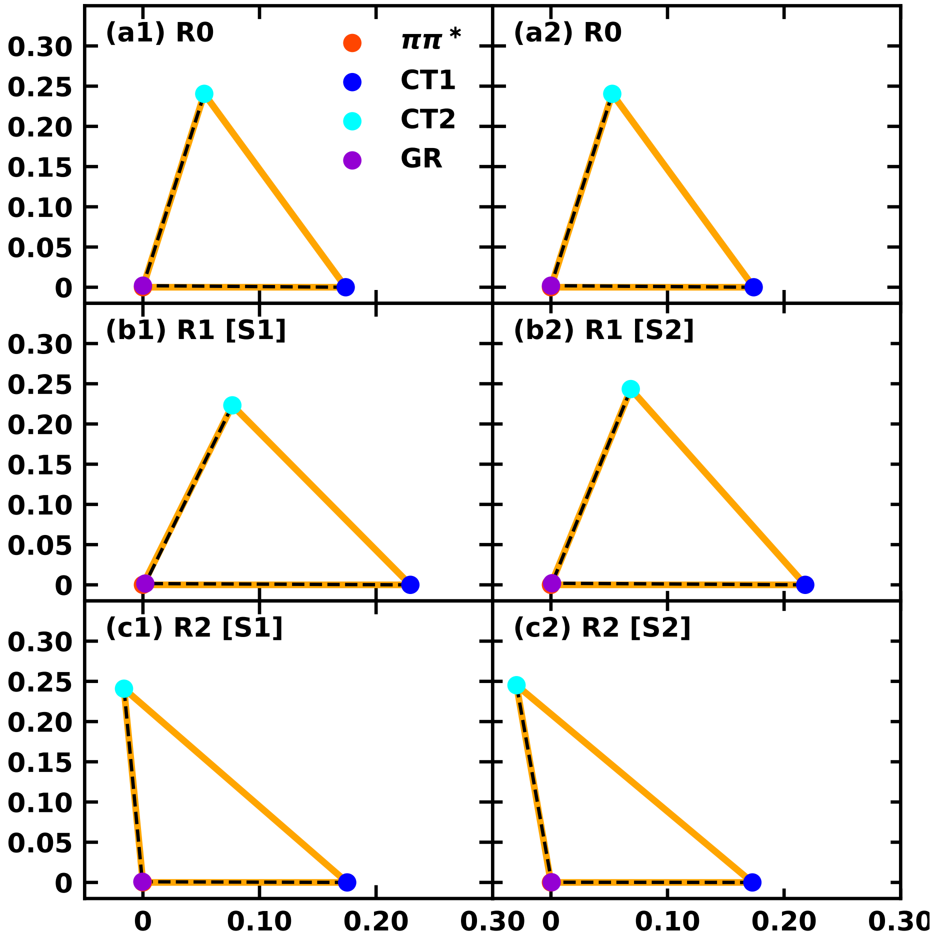

FIG. S1. MSH triangles of R0, R1, and R2 configurations using Schemes #1 and #3. Configuration R0 is the ground state optimized structure in the gas phase, R1 is the more correlated case, and R2 is the anticorrelated case. Conformations can be found in Fig. 1 of the main text.

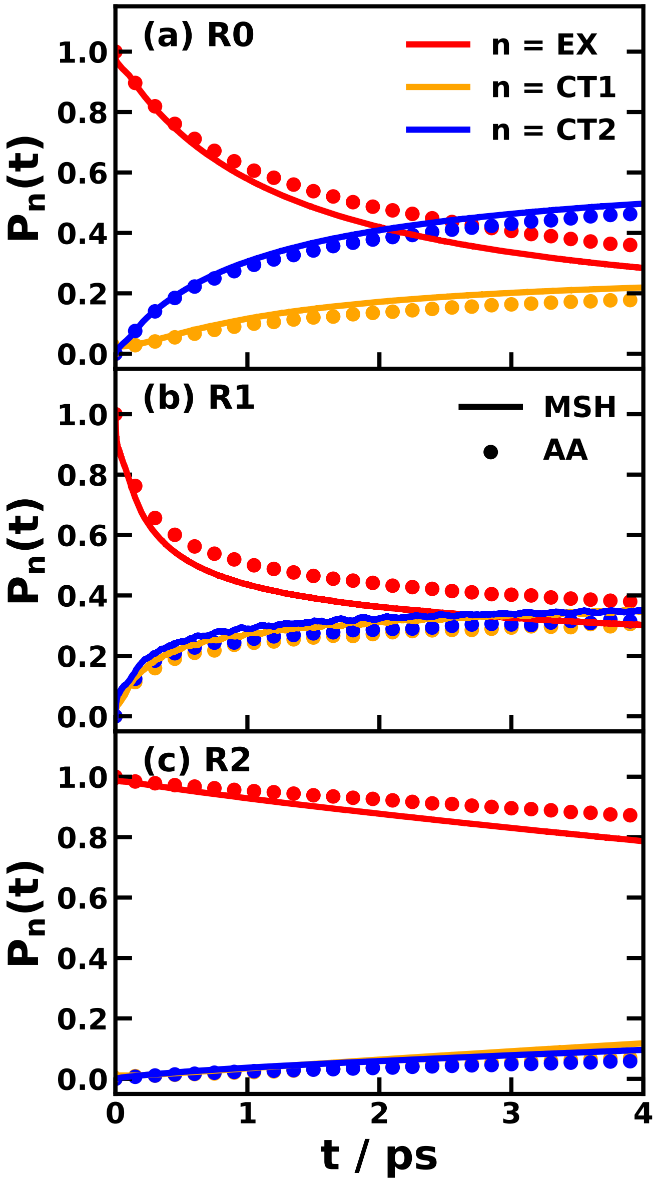

FIG. S2. Photoinduced nonadiabatic dynamics obtained with all-atom Hamiltonian (AA, dot) and multisate harmonic model (MSH, solid line) for conformations R0, R1, and R2 of the MPe/TCNE2 trimer, where the population of bright EX state (red) to CT1 (orange) and CT2 (blue) states. The nonadiabatic dynamics are simulated with symmetrical quasiclassical model (SQC) with triangle window functions and $\gamma = 0.333$ at 300 K. All results correspond to Scheme 1.

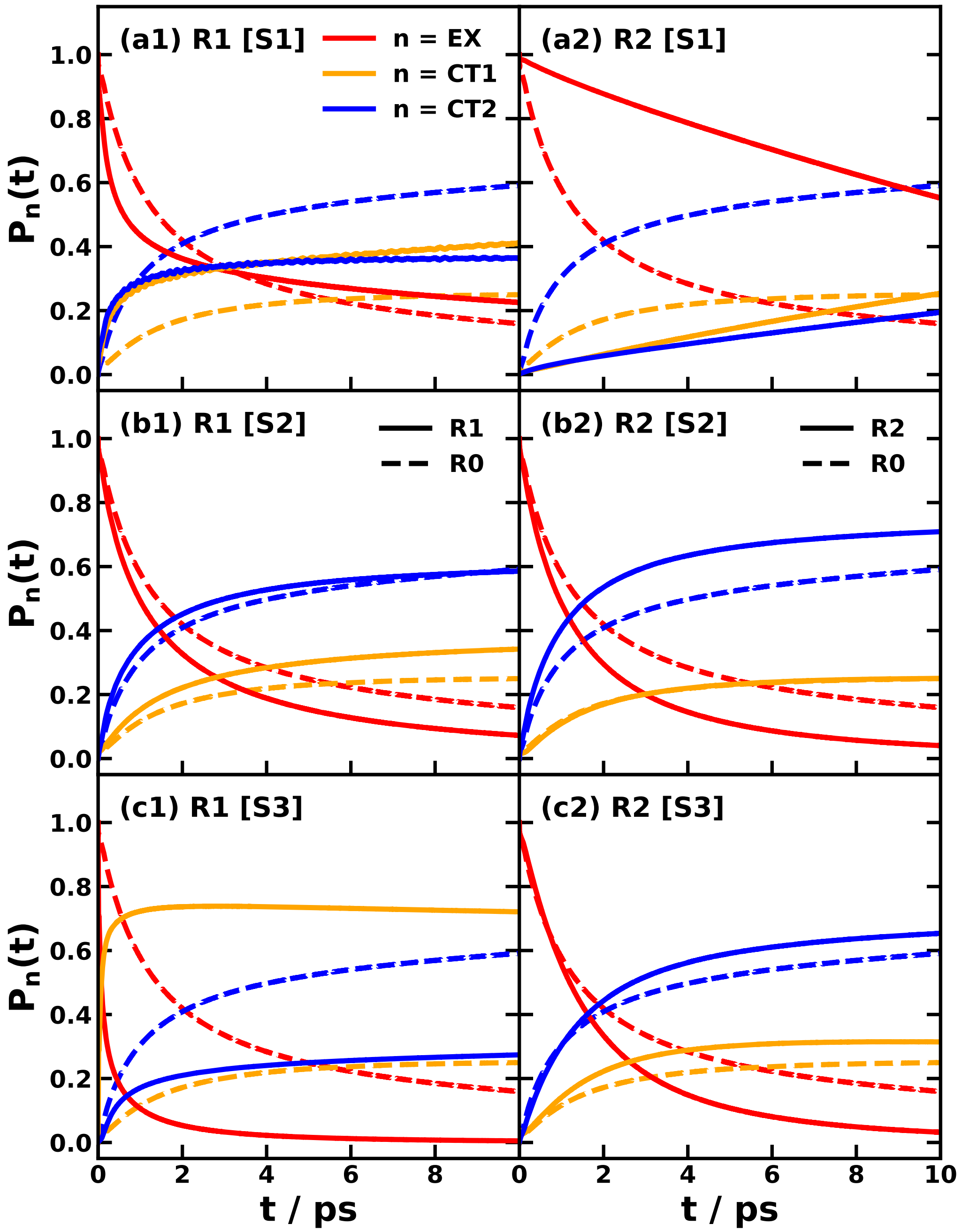

FIG. S3. Photoinduced nonadiabatic dynamics obtained with the MSH models (solid line) of Schemes 1–3 (rows a, b, and c) for conformations R1 (left column) and R2 (right column) of the MPe/2TCNE trimer, compared with the MSH model of conformation R0 (dashed line), where the population of bright EX state (red) transfers to CT1 (orange) and CT2 (blue) states. The nonadiabatic dynamics are simulated via SQC with $\gamma = 0.333$ at 300 K.

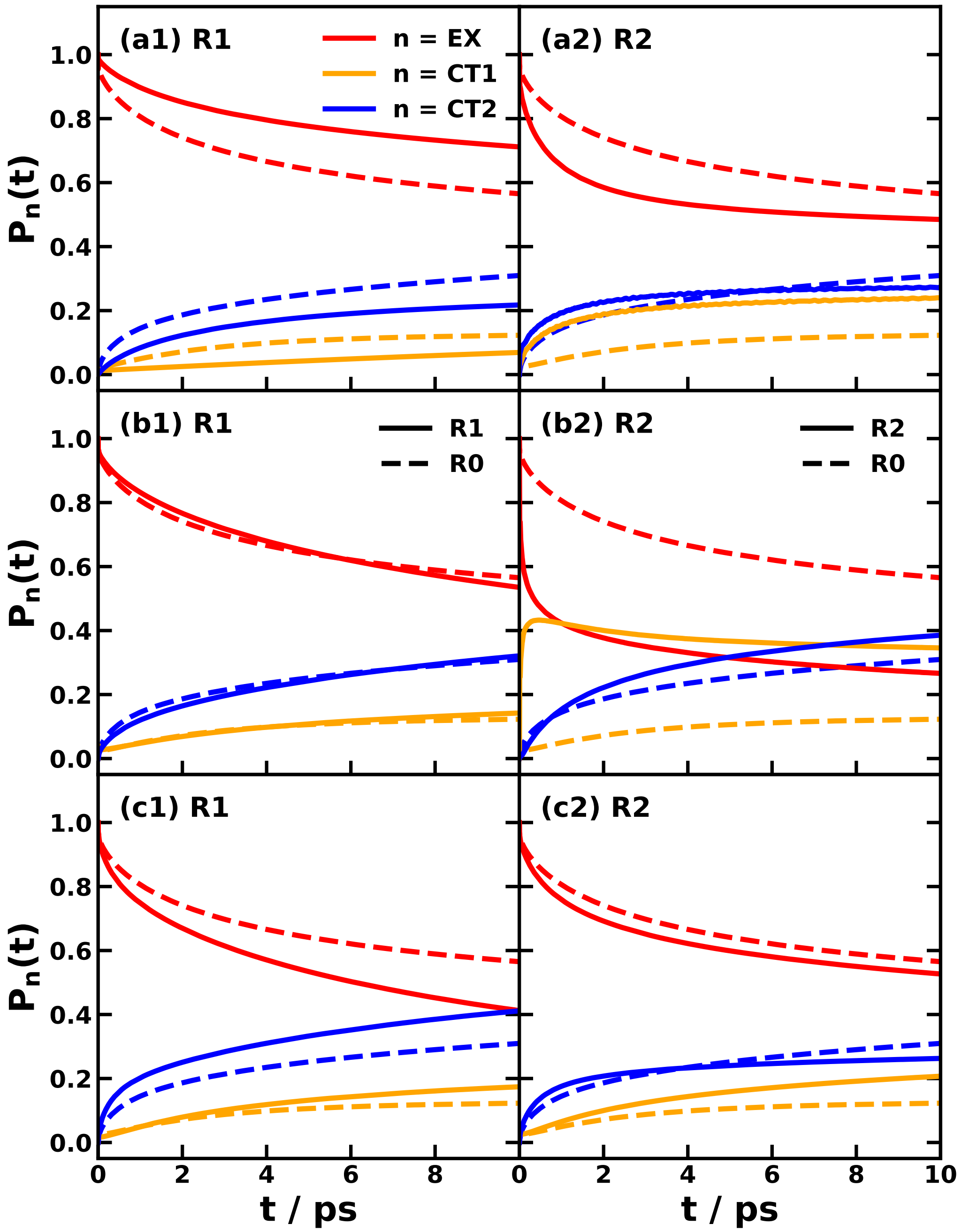

FIG. S4. Photoinduced nonadiabatic dynamics obtained with the MSH models (solid line) of Schemes 1–3 (rows a, b, and c) for conformations R1 (left column) and R2 (right column) of the MPe/2TCNE trimer, compared with the MSH model of conformation R0 (dashed line), where the population of bright EX state (red) transfers to CT1 (orange) and CT2 (blue) states. The nonadiabatic dynamics are simulated via Ehrenfest mean-field (MF) at 300 K.

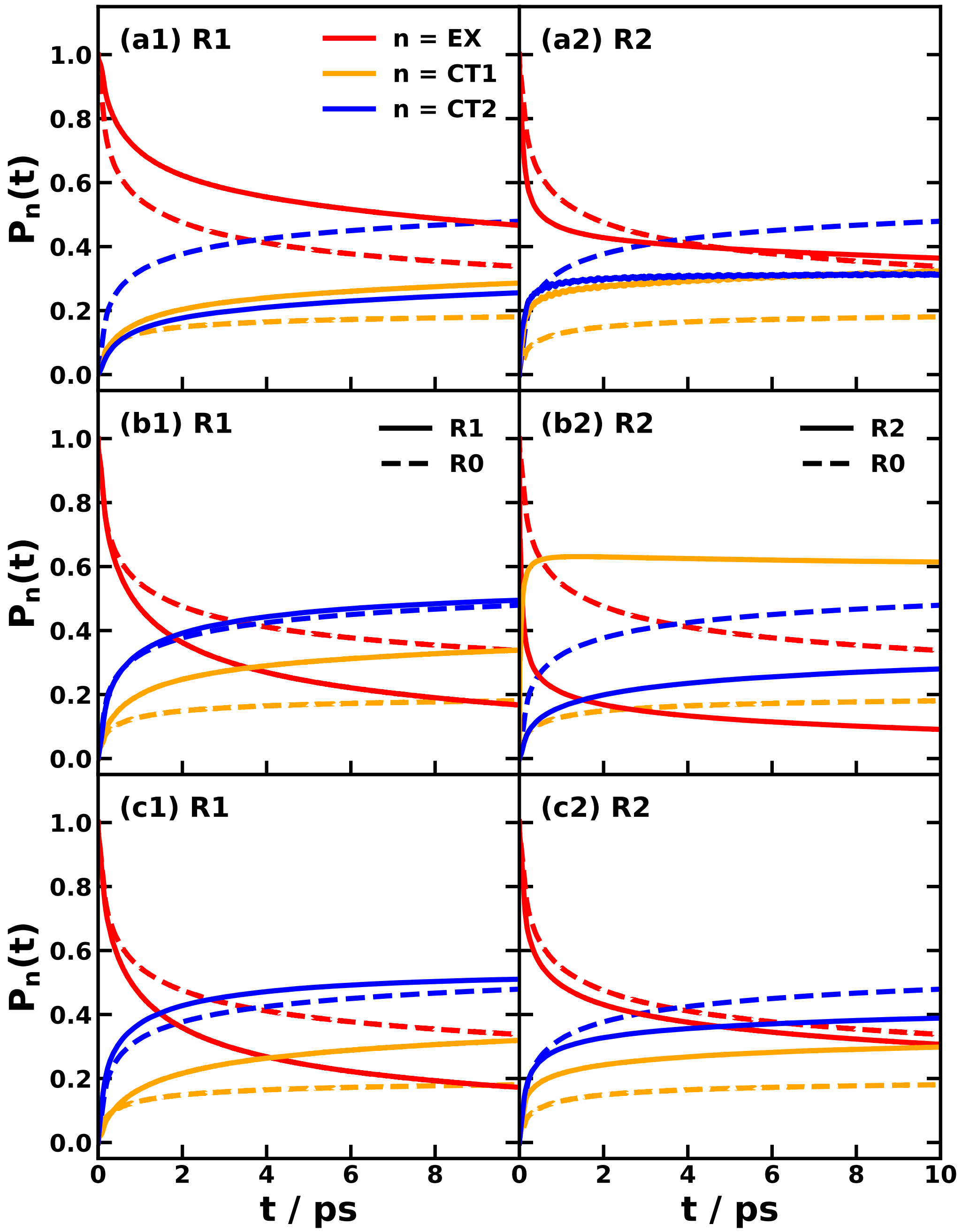

FIG. S5. Photoinduced nonadiabatic dynamics obtained with the MSH models (solid line) of Schemes 1–3 (rows a, b, and c) for conformations R1 (left column) and R2 (right column) of the MPe/2TCNE trimer, compared with the MSH model of conformation R0 (dashed line), where the population of bright EX state (red) transfers to CT1 (orange) and CT2 (blue) states. The nonadiabatic dynamics are simulated via resolution-of-identity linearized semiclassical #1 (RI-LSC1) with $\gamma = 0.5$ at 300 K.

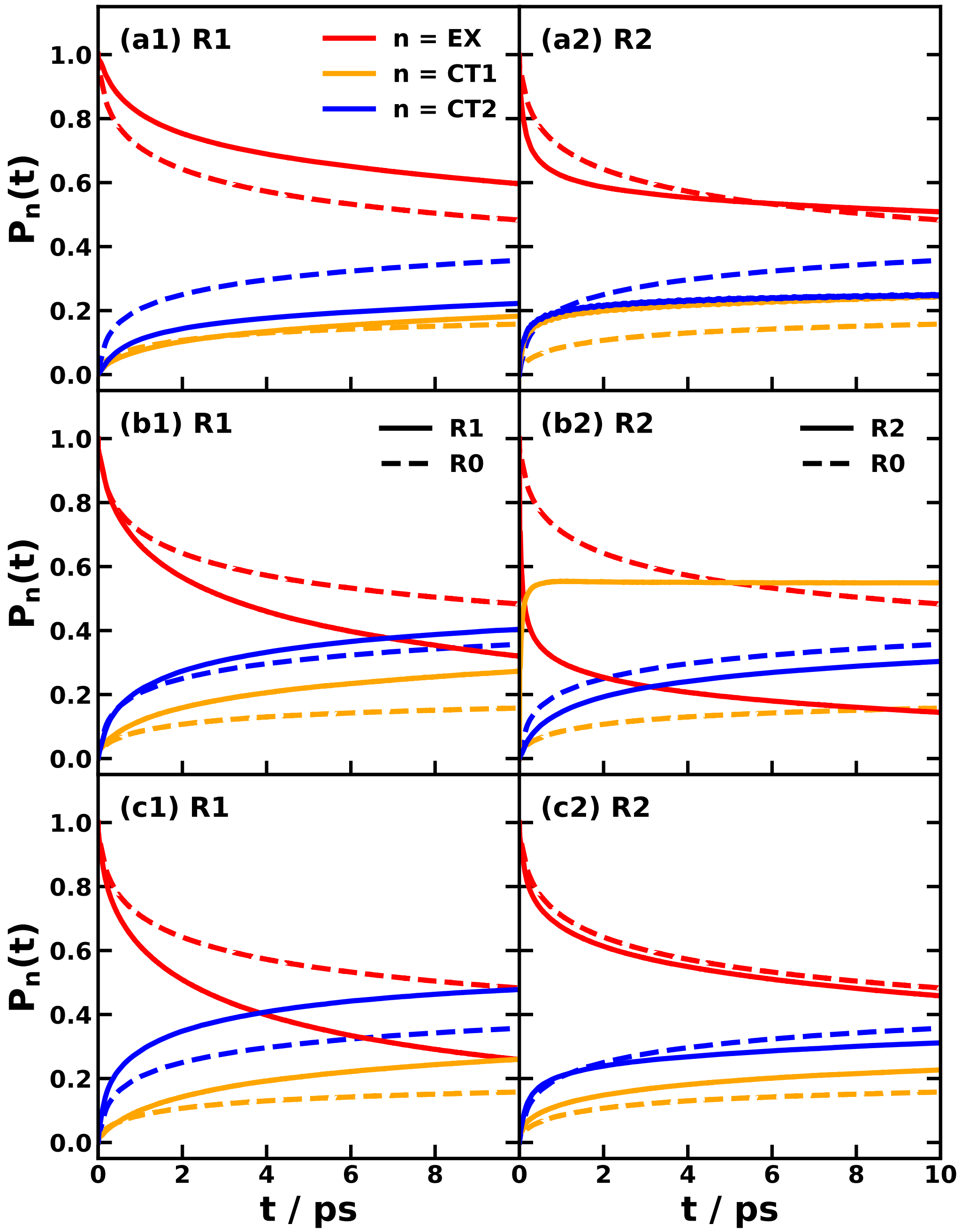

FIG. S6. Photoinduced nonadiabatic dynamics obtained with the MSH models (solid line) of Schemes 1–3 (rows a, b, and c) for conformations R1 (left column) and R2 (right column) of the MPe/2TCNE trimer, compared with the MSH model of conformation R0 (dashed line), where the population of bright EX state (red) transfers to CT1 (orange) and CT2 (blue) states. The nonadiabatic dynamics are simulated via resolution-of-identity linearized semiclassical #2 (RI-LSC2) with $\gamma = 0.5$ at 300 K.

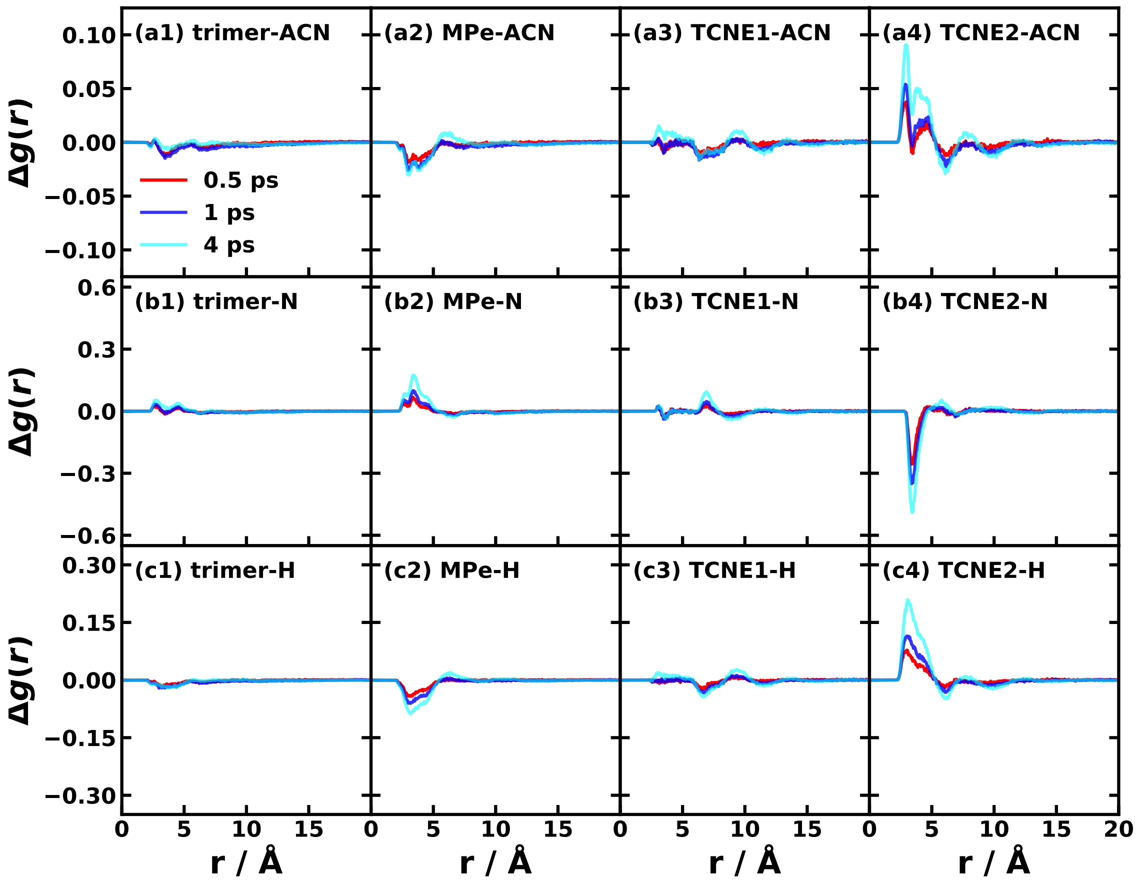

FIG. S7. Nonequilibrium solute-solvent radial distribution function (RDF) evolution $\Delta g(r)$ of conformation R0 of MPe/2TCNE trimer at times of $t = 0.5$ (red), 1 (blue), and 4 ps (cyan) with respect to the initial photoexcitation time $t = 0$. The four columns correspond to the surface of the whole trimer (col. 1), MPe donor (col. 2), TCNE No. 1 (col. 3), and TCNE No. 2 (col. 4) solute with respect to the different solvent parts, respectively; the three rows correspond to all ACN solvent atoms (row a), the N atoms of ACN (row b), and the H atoms of ACN (row c), respectively. The nonadiabatic dynamics are simulated with the all-atom Hamiltonian (scheme 1) using SQC dynamical method with the triangle window at 300 K.

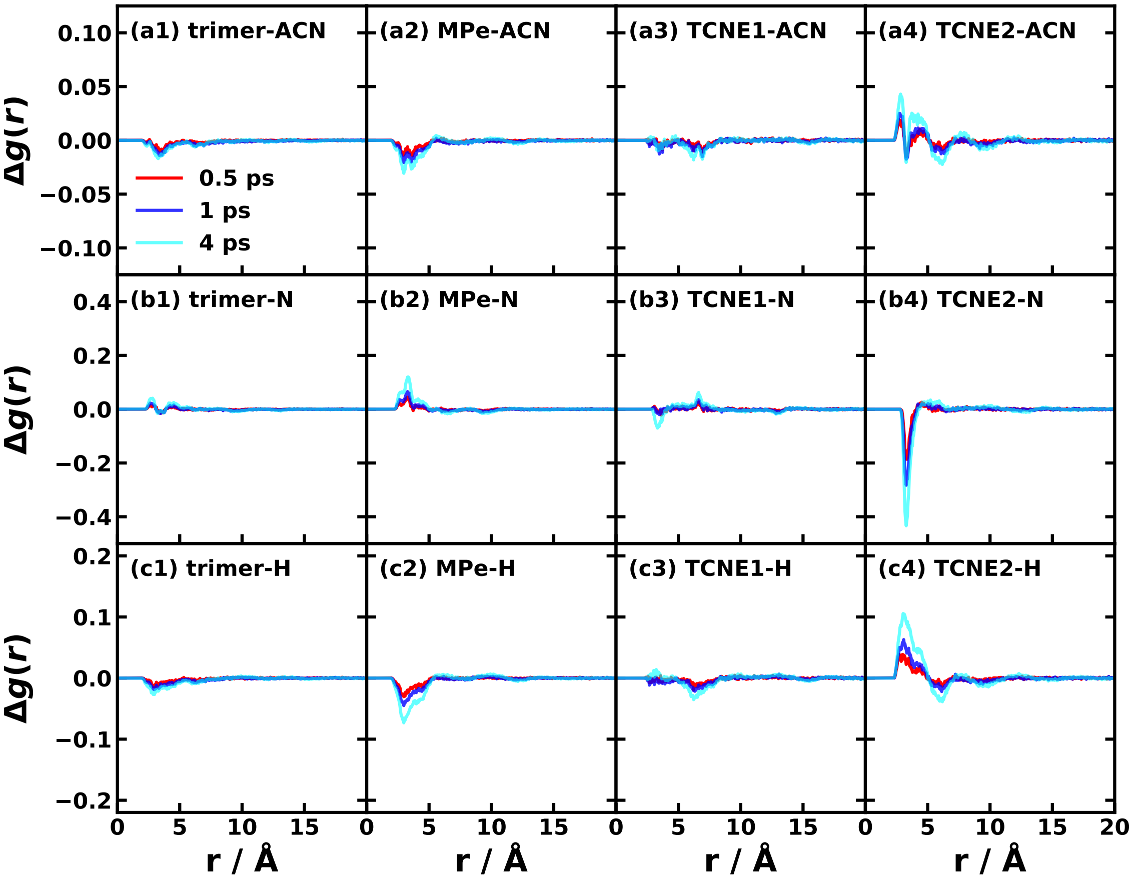

FIG. S8. Nonequilibrium solute-solvent radial distribution function (RDF) evolution $\Delta g(r)$ of conformation R2 of MPe/2TCNE trimer at times of $t = 0.5$ (red), 1 (blue), and 4 ps (cyan) with respect to the initial photoexcitation time $t = 0$. The four columns correspond to the surface of the whole trimer (col. 1), MPe donor (col. 2), TCNE No. 1 (col. 3), and TCNE No. 2 (col. 4) solute with respect to the different solvent parts, respectively; the three rows correspond to all ACN solvent atoms (row a), the N atoms of ACN (row b), and the H atoms of ACN (row c), respectively. The nonadiabatic dynamics are simulated with the all-atom Hamiltonian (scheme 1) using SQC with triangle window dynamical method at 300 K.

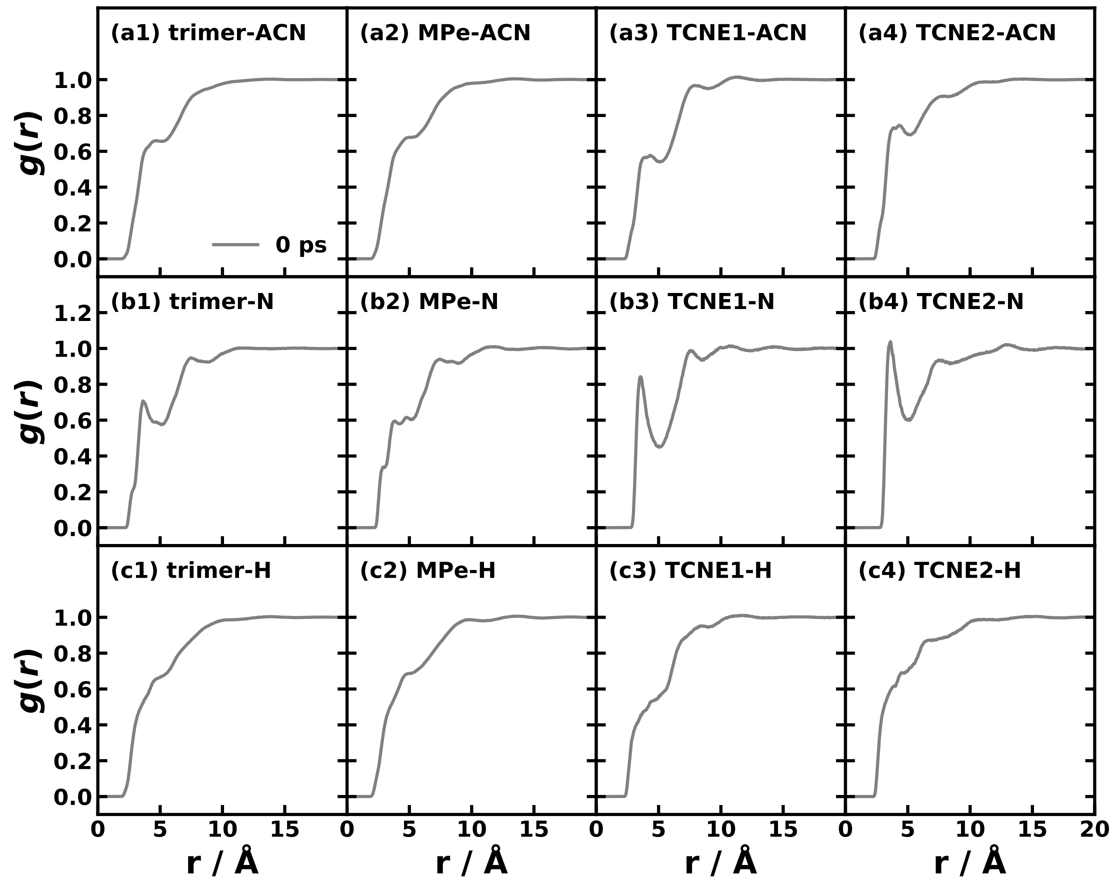

FIG. S9. Equilibrium solute-solvent RDF $g(r)$ of configuration R0 of MPe/2TCNE trimer. The four columns correspond to the surface of the whole trimer (col. 1), MPe donor (col. 2), TCNE No. 1 (col. 3), and TCNE No. 2 (col. 4) solute with respect to the different solvent parts, respectively; the three rows correspond to all ACN solvent atoms (row a), the N atoms of ACN (row b), and the H atoms of ACN (row c), respectively. The nonadiabatic dynamics are simulated with the all-atom Hamiltonian (scheme 1) using SQC with triangle window dynamical method at 300 K.

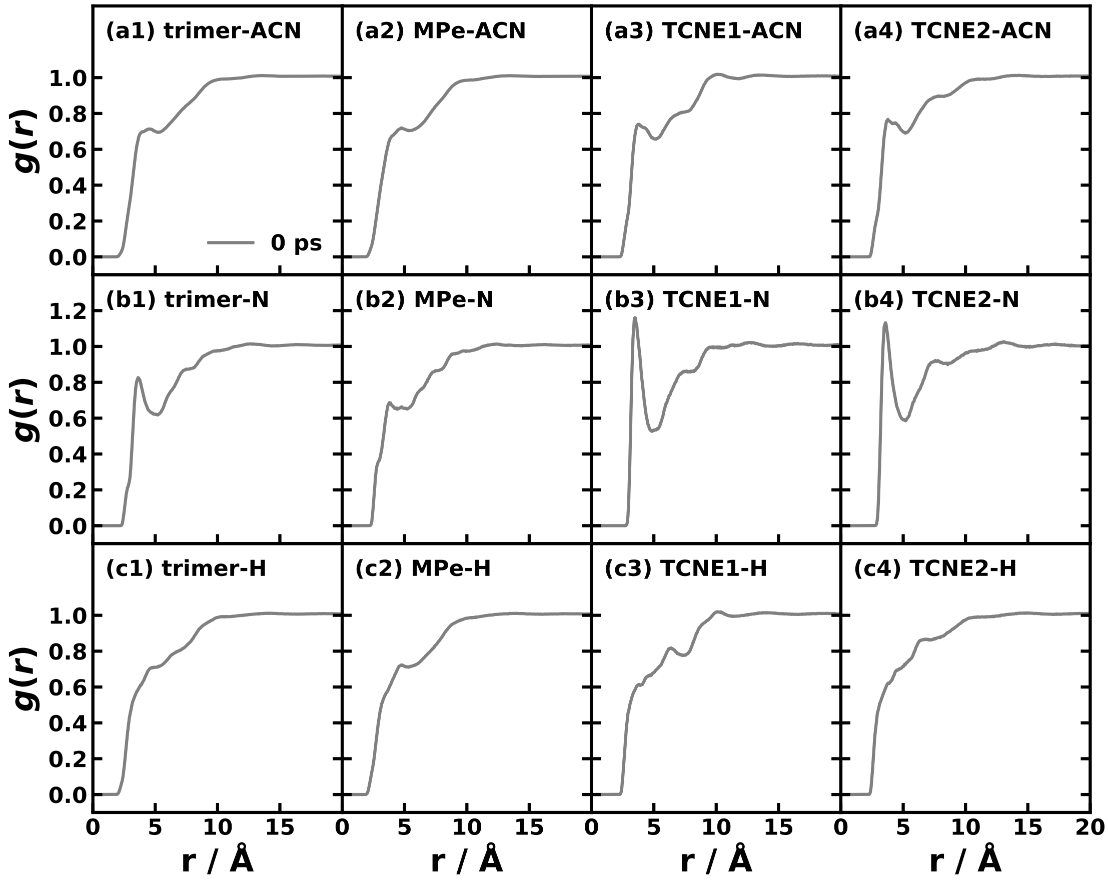

FIG. S10. Equilibrium solute-solvent RDF $g(r)$ of configuration R1 of MPe/2TCNE trimer. The four columns correspond to the surface of the whole trimer (col. 1), MPe donor (col. 2), TCNE No. 1 (col. 3), and TCNE No. 2 (col. 4) solute with respect to the different solvent parts, respectively; the three rows correspond to all ACN solvent atoms (row a), the N atoms of ACN (row b), and the H atoms of ACN (row c), respectively. The nonadiabatic dynamics are simulated with the all-atom Hamiltonian (scheme 1) using SQC with triangle window dynamical method at 300 K.

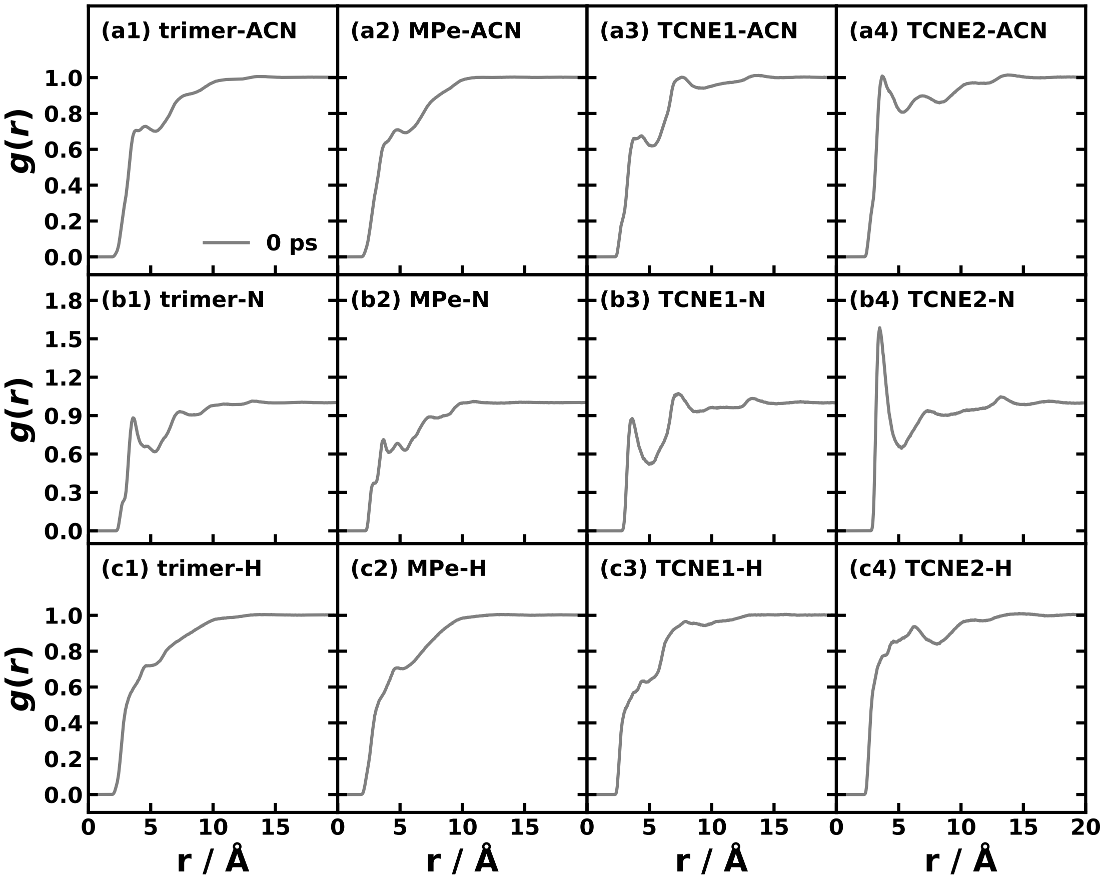

FIG. S11. Equilibrium solute-solvent RDF $g(r)$ of configuration R2 of MPe/2TCNE trimer. The four columns correspond to the surface of the whole trimer (col. 1), MPe donor (col. 2), TCNE No. 1 (col. 3), and TCNE No. 2 (col. 4) solute with respect to the different solvent parts, respectively; the three rows correspond to all ACN solvent atoms (row a), the N atoms of ACN (row b), and the H atoms of ACN (row c), respectively. The nonadiabatic dynamics are simulated with the all-atom Hamiltonian (scheme 1) using SQC with triangle window dynamical method at 300 K.

REFERENCES

[1]M. J. Frisch, G. W. Trucks, H. B. Schlegel, G. E. Scuseria, M. A. Robb, J. R. Cheeseman, G. Scalmani, V. Barone, G. A. Petersson, H. Nakatsuji, *et al.*, "Gaussian16 Revision C.01," Gaussian Inc. Wallingford CT (2016).

[2]J. J. P. Stewart, "Optimization of Parameters for Semiempirical Methods V: Modification of Nddo Approximations and Application to 70 Elements," J. Mol. Model. **13**, 1173–1213 (2007).

[3]A. A. Voityuk and N. Rösch, "Fragment Charge Difference Method for Estimating Donor–Acceptor Electronic Coupling: Application to DNA π-Stacks," J. Chem. Phys. **117**, 5607–5616 (2002).

[4]E. Epifanovsky, A. T. B. Gilbert, X. Feng, J. Lee, Y. Mao, N. Mardirossian, P. Pokhilko, A. F. White, M. P. Coons, *et al.*, "Software for the Frontiers of Quantum Chemistry: An Overview of Developments in the Q-Chem 5 Package," J. Chem. Phys. **155**, 084801 (2021).

[5]C. Adamo and V. Barone, "Toward Reliable Density Functional Methods Without Adjustable Parameters: The PBE0 Model," J. Chem. Phys. **110**, 6158–6170 (1999).

[6]S. Hirata and M. Head-Gordon, "Time-Dependent Density Functional Theory within the Tamm–Dancoff Approximation," Chem. Phys. Lett. **314**, 291–299 (1999).

[7]W. D. Cornell, P. Cieplak, C. I. Bayly, I. R. Gould, K. M. Merz, D. M. Ferguson, D. C. Spellmeyer, T. Fox, J. W. Caldwell, and P. A. Kollman, "A second generation force field for the simulation of proteins, nucleic acids, and organic molecules," J. Am. Chem. Soc. **117**, 5179–5197 (1995).

[8]J. Wang, R. M. Wolf, J. W. Caldwell, P. A. Kollman, and D. A. Case, "Development and Testing of a General Amber Force Field," J. Comput. Chem. **25**, 1157–1174 (2004).

[9]Z. Hu and X. Sun, "All-Atom Nonadiabatic Semiclassical Mapping Dynamics for Photoinduced Charge Transfer of Organic Photovoltaic Molecules in Explicit Solvents," J. Chem. Theory Comput. **18**, 5819–5836 (2022).

[10]T. Darden, D. York, and L. Pedersen, "Particle Mesh Ewald: An Nlog(N) Method for Ewald Sums in Large Systems," J. Chem. Phys. **98**, 10089–10092 (1993).

[11]G. Ciccotti and J. P. Ryckaert, "Molecular Dynamics Simulation of Rigid Molecules," Comput. Phys. Rep. **4**, 346–392 (1986).
